# A Dim Space Target Detection and Track Association Method for Dense Stellar Backgrounds

**DOI:** 10.3390/s26165294

**Published:** 2026-08-21

**Authors:** Cheng Jiang, Zhixia Yang, Zhongqi Ma, Chiming Tong, Jinshen Wang

**Affiliations:** 1 Beijing Institute of Space Mechanics & Electricity, Beijing 100094, China; mazhongqi@cast508.cn (Z.M.); tongcm.11s@igsnrr.ac.cn (C.T.); 2 School of Astronautics, Beihang University, Beijing 100191, China; 15525147861@163.com (Z.Y.); wangjinshen@buaa.edu.cn (J.W.)

**Keywords:** space target detection, track association, inter-frame registration, spectrum phase shift reconstruction, image difference fusion, directional track association

## Abstract

In space target surveillance missions, effective detection of dim space targets remains a challenge due to dense star interference and the random nature of target motion. To quickly and accurately extract dim space targets from complex star backgrounds, this paper proposes a dim space target detection and track association method for dense star backgrounds. This paper analyzes various features of the target and background from a combined spatiotemporal perspective, including three main stages. First, inter-frame registration is used to filter out bright stars, followed by connected domain post-processing, which simplifies the star map background while enhancing the signal-to-noise ratio of dim targets. Secondly, an image difference fusion coarse processing module is proposed. The reconstructed multi-impulse function is derived from the spectral phase difference to estimate the displacement parameters of different components, after which the image difference fusion is designed to obtain candidate targets. Third, a directional track association algorithm is designed, with the candidate targets as the center and the motion parameters as thresholds, narrowing the association range to a fan-shaped region. This enables fast detection of target tracks while removing excess false alarms. The experimental results on four datasets demonstrate that this method outperforms traditional baseline methods in terms of target detection and localization accuracy.

## 1. Introduction

With the rapid development of the space industry, the number of spacecraft launched into space by humans has been continuously increasing, leading to a large amount of defunct spacecraft and debris, which pose a serious threat to the safety of on-orbit spacecraft [1,2,3]. Therefore, there is an urgent need to identify, detect and track these objects to ensure the safety of active spacecraft and accommodate future space development needs. Due to the significant distance between space objects and observation equipment, the targets appear as only a few pixels in the images, lacking clear geometric texture features [4,5,6]. As the number of stars in the field of view increases, the main challenges in detecting space targets include the following. The dense distribution of bright and faint stars with similar morphology to space objects creates strong interference. The targets are weak and lack distinctive features, making them easily lost in the deep-space background. In addition, their motion directions and speeds vary across image sequences.

At present, traditional detection methods mainly utilize the spatial and temporal characteristics of the targets for detection. Image filtering methods estimate the image background based on gray information in pixels but are sensitive to noise. Human vision-based methods that combine spatiotemporal contrast features struggle to distinguish stars that are similar to the target, resulting in many false alarms. Methods such as low-rank sparse representation and optical flow require complex computations, which do not meet real-time requirements. Methods such as matched filtering require knowledge of target morphology or motion priors, and are not robust for detecting dim targets. The multi-frame image differencing method is prone to issues such as incorrect matches, target misses, and residual faint stars in dense stellar backgrounds. Multi-level hypothesis testing and rapid trajectory prediction methods require constructing a selection tree and performing large-scale blind detection for each frame through inter-frame motion prediction, leading to slow detection speeds, inaccurate elimination, and low detection accuracy. In summary, the core of the detection process is to first recognize the target and then locate it across multiple frames. Improvements are still needed in noise sensitivity, star suppression, robust detection, and real-time performance.

It should be emphasized that phase correlation itself is a classical technique for estimating translational displacement between images. Existing phase-correlation-based methods mainly focus on estimating a single dominant global displacement between two frames, and their performance may degrade when multiple motion components coexist or when weak target-related peaks are submerged by strong stellar-background responses. In contrast, the novelty of this work is not the use of phase correlation alone, but the proposed spectrum phase shift screening and reconstruction strategy. Specifically, multiple candidate correlation peaks are extracted from the phase-correlation map, background and target-related peaks are separated in the displacement space, abnormal single-frame displacement estimates are rejected by sequence-level consistency screening, and stable background/target motion parameters are reconstructed for subsequent image difference fusion and directional association.

Aiming at the problems of the high false alarm rate and low detection rate of weak targets in traditional multi-frame detection algorithms under dense stellar backgrounds, this paper conducts multi-stage stellar filtering, dim target detection, and directional track extraction based on the characteristics of targets and stars. Due to severe stellar interference, image preprocessing in the initial stage will complicate the background and lead to missed target detection. Therefore, this paper performs connected domain post-processing after filtering out bright stars, which can simplify the star map background while enhancing the target signal-to-noise ratio. In the target detection stage, this paper proposes an image differential fusion coarse processing module based on spectral phase shift reconstruction, which uses the reconstructed multi-impulse function to accurately estimate the motion parameters of different components, and then obtains candidate targets in the coarse processing module, effectively reducing the number of tracks. In the track association stage, this paper designs a directional track correlation algorithm based on motion parameters and candidate targets, which narrows the search area to a fan-shaped range, accurately and quickly detects the target track while removing redundant false alarms. The main research contributions of this paper are summarized as follows.

- Effective star suppression is achieved in multiple stages based on star brightness. Bright stars are removed through registration between adjacent frames, while faint stars are eliminated using image differencing and fusion in coarse processing module. This ensures precise suppression of stars with varying brightness levels.
- A spectrum phase shift screening and reconstruction strategy is proposed to extend conventional phase-correlation displacement estimation from single dominant motion estimation to multi-component motion analysis in dense stellar backgrounds. It ranks candidate peaks, separates background and target-related responses, and reconstructs stable displacement sequences through inter-frame consistency screening. Based on the reconstructed motion parameters, the image difference fusion coarse processing module suppresses faint stellar interference and effectively extracts candidate targets.
- The directional track association algorithm designed in this paper, using candidate targets as trajectory points and motion parameters as threshold values, can narrow the association range to a fan-shaped area. This allows for precise detection of target trajectories while eliminating false alarms.

The rest of the article is presented as follows. Related work is outlined in Section 2. The proposed method is detailed in Section 3. Section 4 presents the experimental results and discusses the state-of-the-art performance. Finally, the conclusions and future works are provided in Section 5.

## 2. Related Work

In recent years, many methods have been proposed for space target detection. The existing methods are mainly divided into three categories: star map recognition methods, detection and tracking methods as well as deep learning methods.

### 2.1. Star Map Recognition Methods

The star map recognition algorithm can be primarily divided into subgraph isomorphism methods and pattern recognition methods in terms of recognition strategies [7].

The subgraph isomorphism method is based on graph theory, treating the star map as an undirected graph to determine whether the target star map is isomorphic to a part of a known template star map. Wang et al. proposed a fast and robust star identification algorithm based on a hash map, which maps triangle features to integers and builds a hash table [8]. They designed a double-triangle verification method and an optimized feature selection strategy, achieving a high identification rate. Due to the inefficiencies and reliability issues of the polygon algorithm and match group algorithm, Liu et al. proposed a star identification algorithm based on the simplest general subgraph [9]. This algorithm evaluates the validity of different subgraphs by developing an analytical model for selection and demonstrates strong robustness against noise and false stars. Traditional star identification algorithms rely solely on geometric position information, requiring a high number of imaged stars. Niu et al. proposed a star identification algorithm using color ratio information to solve this problem [10]. By introducing color ratio information as a recognition feature, this algorithm not only reduces the number of false matching star pairs but also accelerates the search process. The subgraph isomorphism method has high structural robustness and accuracy, but when the template library is large, it faces challenges in computational complexity and matching efficiency.

The pattern recognition method posits that each star has a unique pattern formed by its companion stars, generating star patterns based on the spatial relationships between observed stars and constructing a matching library. To meet the requirements of a large field of view and low sensitivity, Li et al. proposed an improved grid algorithm that effectively increases the probability of finding correct neighboring stars and reduces pattern noise by constructing star pair patterns and two-dimensional angular distance features [11]. Due to the sensitivity of the radial pattern to noise and the low efficiency in eliminating spurious matches, Liu et al. developed a novel star identification algorithm based on the recommended radial pattern [12]. By introducing the apriori algorithm to mine the association rules between stars and neighboring stars, the algorithm generates recommended radial patterns and demonstrates strong robustness against position noise, brightness noise, and false stars. Due to the complexity of computation and storage, there are few studies that utilize angular distance matching for direct identification. Wei et al. proposed a graph algorithm based on angular distance matching score transfer [13], which uses a graph data structure where nodes store angular distance matching scores and transfer scores through edge weights, overcoming the limitations of incomplete utilization of matching results. The pattern recognition method is flexible and efficient, but it typically requires a higher number of true stars in the field of view, and the quality of feature selection directly affects the accuracy of recognition.

### 2.2. Detection and Tracking Methods

The detection and tracking methods are based on the correlation characteristics of spatial targets’ morphology and relative positional variations over time for identification. Spatial targets exhibit significant positional changes in image sequences, while background stars remain relatively static or exhibit slow motion. Existing target detection and tracking methods for sequential imagery include: the inter-frame difference method, background mask method, spatio-temporal pipeline filtering method, multi-level hypothesis testing method, low-rank and sparse decomposition method, trajectory correlation method, etc.

To address the impact of noise on detection accuracy, Zhang et al. proposed a three-frame difference algorithm based on mathematical morphology [14], which can effectively detect moving targets while reducing the influence of noise. Linder et al. proposed the Difference Method for onboard space debris detection within ESA’s Optical In Situ Monitor activity [15]. This method performs inter-frame registration using background stars and applies image differencing to suppress stellar interference and enhance moving objects. However, its performance may degrade under complex stellar backgrounds and low signal-to-noise ratio conditions. In 2019, Li et al. proposed a spatiotemporal pipeline multistage hypothesis testing (SPMHT) method [16]. This approach utilizes spatiotemporal-related intersection over union (SrIoU) and pipeline filtering to effectively eliminate background stars and extract candidate points. Subsequently, it prunes candidate trajectories via a tree-like structure, significantly enhancing the accuracy and robustness of space target detection. Under conditions of high target diversity and significant interference, Lin et al. proposed a robust space target detection algorithm based on target characteristics (MLTC) [17]. The algorithm first utilizes local saliency to generate center point proposals, then designs three features based on the distribution characteristics of the targets and computes saliency maps for detection. This method demonstrates superior performance in accuracy, false alarm rate, and other key metrics. In 2022, Jiang et al. proposed a faint space debris detection algorithm based on small aperture telescopes [18]. By integrating wavelet decomposition and guided filtering to suppress a large number of star points, and subsequently utilizing low-rank and sparse decomposition to solve the target matrix, this method enables effective detection of faint debris in small-aperture monitoring systems. To address the presence of numerous stars and noise in star maps that share similar characteristics with the targets to be detected, Qin et al. (2023) proposed a space target detection method based on the frame difference and target characteristics [19]. This algorithm fully integrates the advantages of the frame difference method and multi-scale local target characteristics, enabling further screening of targets and improving detection efficiency and accuracy. To address the challenge of distinguishing between space debris and stars in small-field detection systems, Zhang et al. proposed a real-time star extraction method based on adaptive filtering and multi-frame projection [20]. By integrating techniques such as bad pixel repair, multi-frame projection, and adaptive filtering, this approach enhances the efficiency and accuracy of star recognition and detection. To address the limitations of low signal-to-noise ratio (SNR) stellar suppression algorithms, Zhang et al. (2023) proposed a dim star background suppression method based on recursive moving target indication (RMTI) [21]. This approach first employs RMTI to enhance faint stars and then applies adaptive threshold segmentation to precisely filter them out. The method demonstrates significant improvements in real-time performance and suppression capability for dim star. In 2024, Su et al. proposed a local spatio-temporal registration (LSTR) method for detecting moving space targets [22]. This approach employs local registration to estimate adjacent background motion models, enhances targets by measuring temporal local–central region differences, and further preserves target signals while suppressing residual clutter. Linares et al. analyzed the detectability of potential collision objects using onboard optical sensors such as star trackers [23]. Their study demonstrated that faint and fast-moving objects are difficult to detect reliably in low-SNR conditions, highlighting the necessity of multi-frame integration and temporal information utilization.

Infrared moving point target detection, as a critical technology, has been widely applied in space surveillance, military, and aerospace fields. Numerous related methods are also being actively utilized and developed in this domain. To achieve higher detection rates and lower false alarm rates, Du et al.(2020) proposed a spatio-temporal local difference measure (STLDM) algorithm for detecting infrared moving small targets [24]. By leveraging the central grayscale variation and surrounding grayscale variation of moving small targets in the 3D spatiotemporal domain, this method demonstrates superior performance across diverse scenarios. To address the challenges of infrared dim and small target detection in complex scenes, Hu et al. proposed a novel multi-frame spatial–temporal patch-tensor (MFSTPT) model [25]. By constructing a new tensor framework through spatial–temporal simultaneous sampling and incorporating weighted prior information, this method achieves significant improvements in detection accuracy and background suppression despite increased computational time costs. Considering the background characteristics and spatiotemporal trajectory continuity of targets, Luo et al. proposed a clustering and tracking-guided spatial–temporal completion (CTSTC) model by constructing a 3D spatial–temporal prediction tensor [26]. By integrating an improved K-means clustering algorithm and Bayesian-inference-derived tracking regularization, this approach synchronously enhances target detectability and background suppression capability. In summary, most existing detection and tracking methods are jointly utilized in multi-stage coarse-to-fine processes to detect spatial moving targets, thereby improving the detection accuracy of dim space targets.

### 2.3. Deep Learning Methods

Due to noisy labels caused by false alarms, networks may overfit and lose their ability to detect debris. Li et al. introduced label noise learning into space debris detection and proposed a novel learning paradigm called “Co-correcting” to overcome the effects of noisy labels [27]. To address the interference caused by complex backgrounds and improve the signal-to-noise ratio of images, Liu et al. proposed a background suppression-based star map enhancement algorithm called MBS-Net in 2023 [28]. The network consists of three parts: the background information estimation stage, multi-level U-Net cascade module, and recursive feature fusion stage, achieving competitive performance. Due to the limited imaging size of space debris and the unknown motion characteristics, Tao et al. proposed a space debris saliency detection algorithm (SDebrisNet) [29]. This algorithm utilizes convolutional neural networks to consider the spatial–temporal data of sequential images, demonstrating good robustness for detecting motion space debris with low signal-to-noise ratios. In response to the differential apparent motion speeds, scales, and morphological characteristics of targets, Guo et al. proposed the multiframe spatio-temporal attention motion-adaptive network (MSAMNet) in 2025. By integrating the adaptive attention feature enhancement module and the spatio-temporal dynamic motion-aware module, this network strengthens the representation capability of joint spatio-temporal features, and significantly improves the detection performance of robust dim and moving space targets against complex backgrounds [30]. To address the detection of small and dim space targets in deep-space observation scenarios, Ren et al. proposed a spatio-temporal collaborative detection model named TESNet [31]. The model adopts a lightweight 3D convolutional backbone, a dual-branch feature extraction structure, and a gated dual-attention fusion module to achieve adaptive fusion of spatial and temporal information, thereby effectively enhancing the perception and detection capability for small and dim targets. However, due to the lack of learnable information, deep learning methods for space target detection still need improvement and development.

## 3. Methods

**Figure 1:**
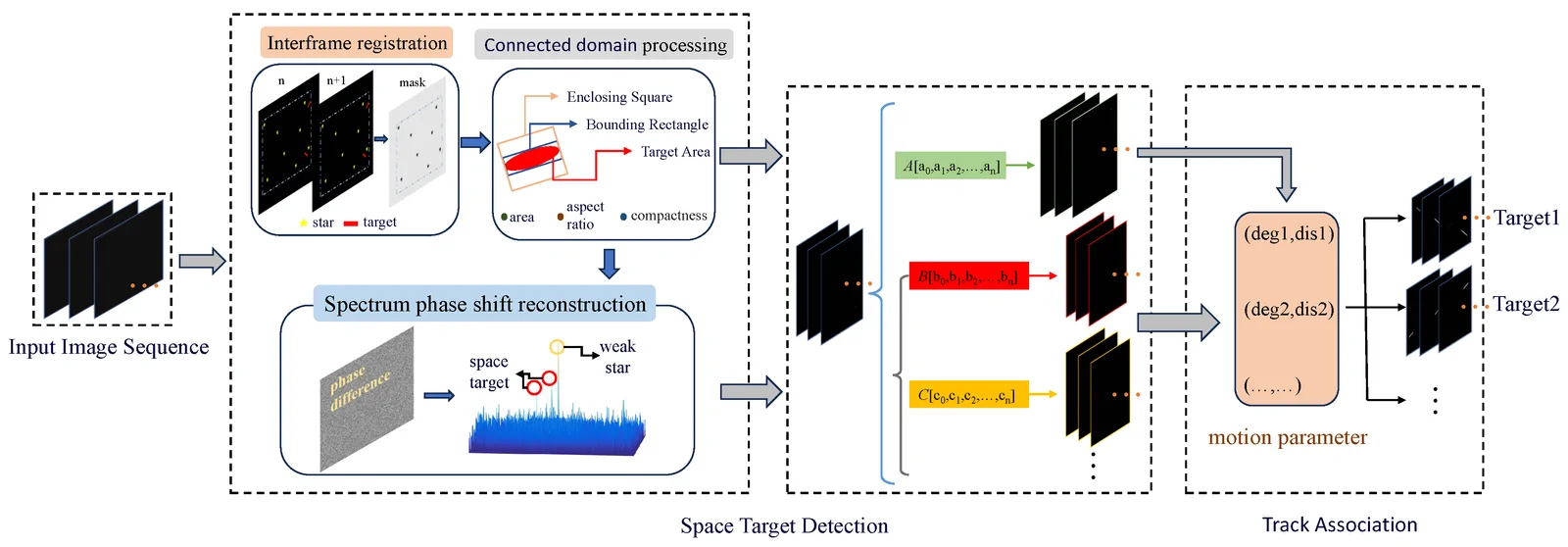
Framework of the proposed method. (The arrows indicate the processing flow, and the dashed boxes represent different processing stages. In the connected-domain processing stage, the colored dots indicate the three filtering criteria, including area, aspect ratio, and compactness).

To quickly and accurately extract faint spatial targets from complex star fields, we propose a dim space target detection and track association method in dense stellar background. The overall framework of the method is illustrated in Figure 1, consisting of three main stages. First, inter-frame image registration and connected domain post-processing are used to filter out bright stars and enhance the signal-to-noise ratio of the targets. Next, we propose an image difference fusion coarse processing module based on spectral phase shift screening and reconstruction, where the reconstructed multi-impulse functions can accurately estimate the motion parameters of faint stars and various targets. This information is then used for image differencing and fusion to obtain candidate targets. Finally, a directional track association algorithm is designed using the candidate targets as centers and the motion parameters as thresholds. This effectively narrows the target association range, allowing for the detection of real trajectories while eliminating unnecessary false alarms.

### 3.1. Bright Star Removal and Connected Domain Processing

Due to the significant influence of component intensity on spectral phase shift, applying this algorithm in dense bright star fields can easily lead to target drowning. At the same time, registration between sequential image frames is sensitive to changes in grayscale. When lighting conditions vary, the brightness of low-magnitude stars remains stable, allowing for accurate matching, while faint stars are prone to incorrect matching due to lighting influences [32]. Therefore, to effectively suppress stars while preserving faint targets, this paper employs a multi-stage star removal strategy. First, we obtain a common region mask between two frames by aligning adjacent frame images, and then construct a sequence of mask templates. Then, we perform differencing between the template sequence and the original image sequence to remove the bright star interference, simplifying the background. To eliminate residual bright stars after differencing, we apply a 5×5 structuring element for dilation on the mask. Finally, the star-removed sequence images are mapped back to their original positions.

(1) $$\begin{array}{cc} M_{i} & =I_{i}\circ \left(I_{i+1}\to I_{i}\right)\\ \; & =I_{i}\circ R_{i+1} \end{array}$$

(2) $$P_{i+1}=\left[R_{i+1}\circ \left(M_{i}⊕D_{0}\right)\right]\Rightarrow I_{i+1}$$

where $I_{i}\;\left(i=1,2,\ldots ,n\right)$ represents the original image sequence, $I_{i+1}\to I_{i}$ denotes the registration of images from frames $I_{i+1}$ to $I_{i}$, $R_{i}\;\left(i=1,2,\ldots ,n\right)$ refers to the registered image sequence, $M_{i}\;\left(i=1,2,\ldots ,n\right)$ indicates the sequence of mask templates, $\Rightarrow I_{i+1}$ represents the processed images mapped back to their original positions according to $I_{i+1}$, $P_{i}\;\left(i=1,2,\ldots ,n\right)$ is the sequence of images after bright star removal, and ∘ and ⊕ denote the matrix dot product and dilation operations, respectively.

It should be noted that the inter-frame registration module in this work is designed for continuous star-field image sequences acquired under relatively stable observation conditions. In the datasets used in this paper, the dominant background motion between adjacent frames is small translation, and no obvious large-angle rotation or large viewpoint change is observed. Therefore, the registration step mainly compensates for translational background displacement. The fixed dilation operators used in the subsequent candidate extraction stage are not intended to correct large geometric registration errors; instead, they provide limited tolerance to small residual misalignments after registration.

**Figure 2:**
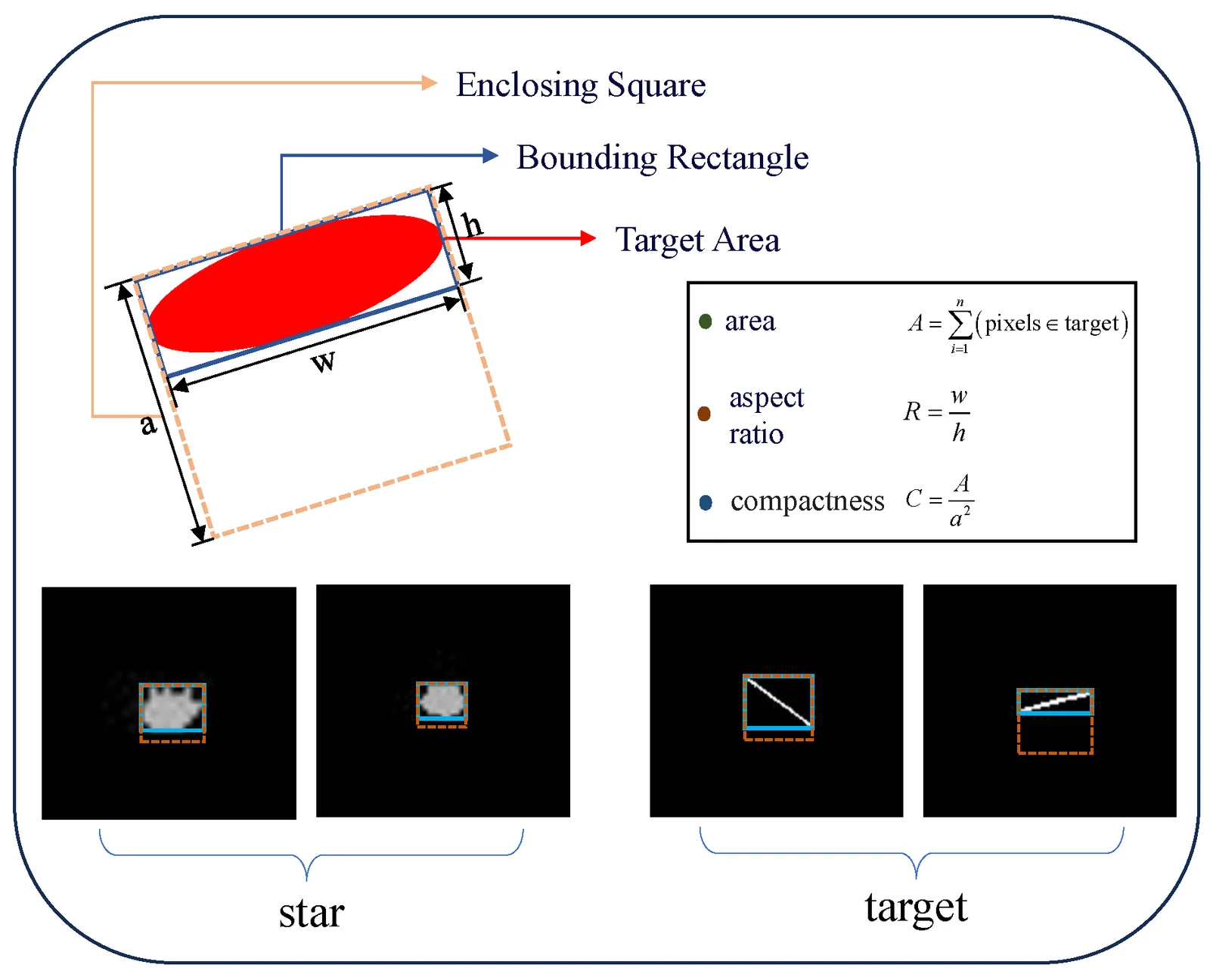
Connected domain post-processing.

To enhance the signal-to-noise ratio of faint targets and eliminate scattered noise, this paper performs connected domain post-processing after removing bright stars. First, we propose an adaptive threshold *T* to obtain the binary image:

(3) T=μ+kσ

where μ and σ are the mean and standard deviation of $P_{i}$, respectively. *k* is an empirical value set to 2.5. Considering the imaging characteristics of optical systems, stars exhibit an approximately symmetric Gaussian distribution, while spatial targets extend in the direction of motion, presenting a striped distribution. Based on these simple spatial characteristics, we propose three shape parameters to suppress noise and enhance the signal-to-noise ratio of the targets (see Figure 2).

(4) $$A=\sum_{i=1}^{n}\left(pixels\in target\right)$$

(5) $$R=\frac{w}{h}$$

(6) $$C=\frac{A}{a^{2}}$$

where *A* denotes the area threshold, which is defined as the total number of foreground pixels within the connected domain; *R* represents the aspect ratio threshold, defined as the ratio between the width and height of the bounding rectangle of the connected domain; and *C* represents the compactness, defined as the ratio between the area of the connected domain and that of its minimum enclosing square. *w* and *h* are the width and height of the bounding rectangle of the connected domain, respectively. And *a* is the side length of the enclosing square of the connected domain. We denote the post-processed sequence of images as $B_{i}\;\left(i=1,2,\ldots ,n\right)$.

The threshold parameters *A*, *R*, and *C* are only used for coarse connected-component filtering during the image post-processing stage. Their purpose is to remove isolated noise, residual stripe artifacts, and connected components whose morphological characteristics are clearly inconsistent with those of real targets. These thresholds are conservatively selected so that only regions that are evidently unlikely to correspond to real targets are removed, while potential target candidates are preserved as much as possible. Therefore, their influence on the final detection performance is relatively limited.

### 3.2. Space Target Detection

**Figure 3:**
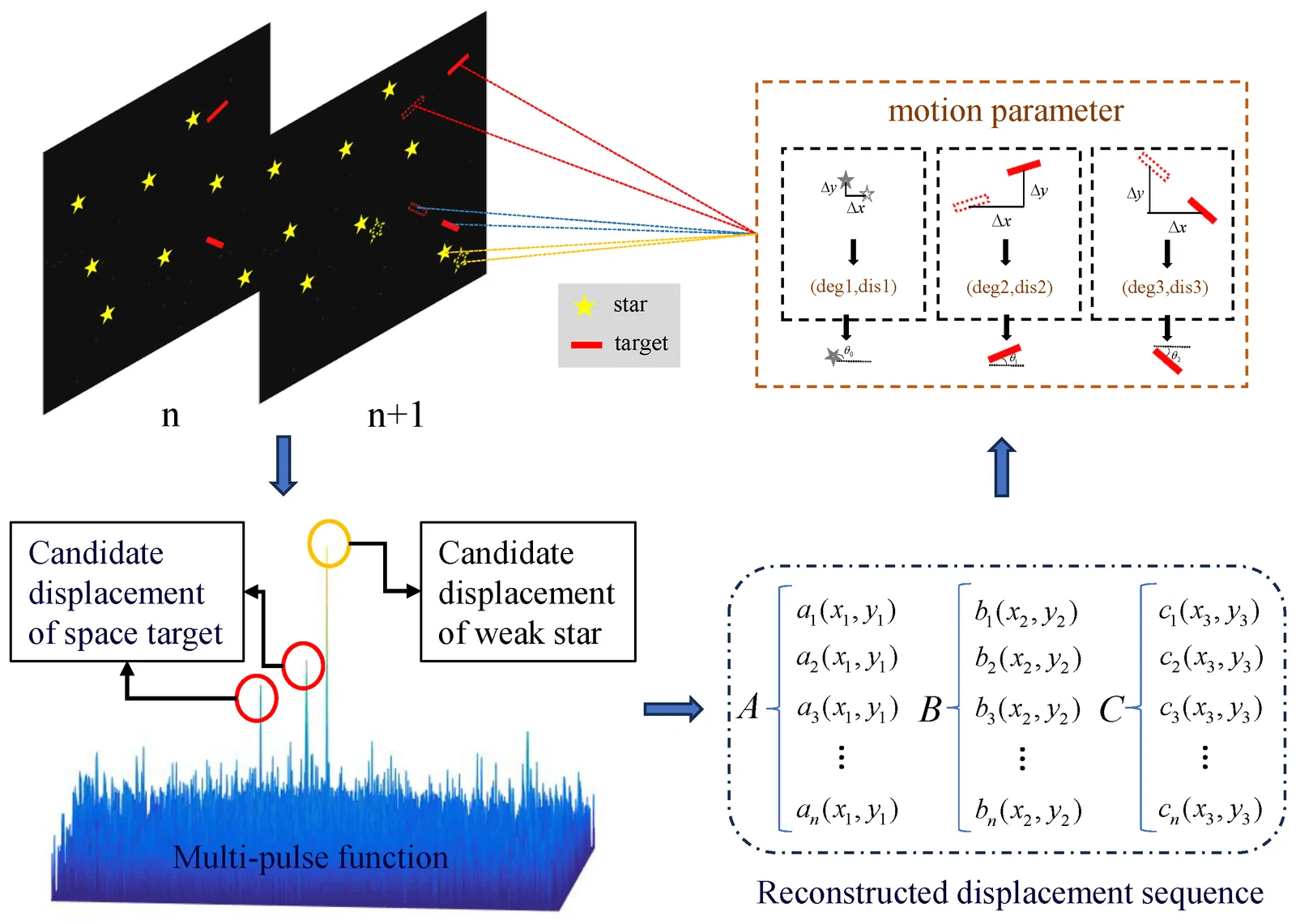
Illustration of the proposed spectrum phase shift screening reconstruction strategy (Arrows indicate the processing flow between different stages).

Using the frequency spectrum phase shift characteristics and inter-frame phase difference method, the relative motion between various components in the sequence of images can be analyzed to obtain specific displacement information [33]. The post-processing of connected domains enhances the visibility of targets, thereby adding reliability to the frequency spectrum phase shift estimation of target motion parameters. However, the accuracy of phase difference calculations can be affected in specific frames due to disturbances such as environmental noise, motion blur, or variations in illumination. Therefore, this paper designs a sequence frame displacement screening and reconstruction method to eliminate abnormal displacement components in specific frames and reconstruct correct displacement information, thereby accurately estimating the motion parameters of both the target and background stars (see Figure 3). As shown in Figure 3, the proposed spectrum phase shift screening and reconstruction module first generates the phase-correlation map between adjacent frames, then extracts and ranks multiple candidate peaks, separates background and target-related displacement responses, and finally reconstructs stable motion parameters through sequence-level consistency screening.

#### 3.2.1. Spectrum Phase Shift Screening Reconstruction

Different from conventional phase correlation methods that directly select the maximum correlation peak as the inter-frame displacement, the proposed method treats the correlation map as a multi-component displacement response. The dominant peak is used to describe the apparent motion of the stellar background, while other significant peaks away from the background peak are regarded as candidate target-motion responses. Since weak target peaks may be unstable in individual frame pairs, sequence-level screening is further introduced to remove abnormal displacement estimates and reconstruct consistent motion sequences. Therefore, the proposed module is a screening-and-reconstruction framework built upon phase correlation, rather than a direct application of standard phase correlation.

The target motion and dim star displacement are both present in the sequence of images, expressed as

(7) $$\left\{\begin{array}{c} B\left(x,y\right)=B_{0}\left(x,y\right)+B_{1}\left(x,y\right)\\ B^{\prime }\left(x,y\right)=B_{0}^{\prime }\left(x,y\right)+B_{1}^{\prime }\left(x,y\right) \end{array}\right.$$

where $B\left(x,y\right),B^{\prime }\left(x,y\right)$ represents two consecutive frames, $B_{0}\left(x,y\right)$ represents the background of faint stars, and $B_{1}\left(x,y\right)$ represents the moving target. Performing Fourier transform on the two consecutive frames results in $F_{1}\left(u,v\right),F_{2}\left(u,v\right)$:

(8) $$F_{1}\left(u,v\right)=F\left\{B\left(x,y\right)\right\}$$

(9) $$F_{2}\left(u,v\right)=F\left\{B^{\prime }\left(x,y\right)\right\}$$

where *x* and *y* are spatial coordinates, and *u* and *v* are frequency domain coordinates, $F\left\{*\right\}$ represents the Fourier transform. Based on the phase shift property of the Fourier transform, calculate the conjugate multiplication of $F_{1}$ and $F_{2}$ to eliminate the amplitude spectrum information in the frequency domain and retain the phase spectrum information to record the phase difference between the two images:

(10) $$R\left(u,v\right)=F_{1}\left(u,v\right)\cdot F_{2}^{*}\left(u,v\right)$$

where R(u,v) denotes the cross-power spectrum, which contains the phase difference information between the two images across all frequencies, and $F_{2}^{*}\left(u,v\right)$ is the complex conjugate of $F_{2}\left(u,v\right)$. To facilitate phase spectrum comparison and extract the displacement information of the target and background, the cross-power spectrum R(u,v) is normalized on an element-wise basis to obtain the normalized cross-power spectrum $R_{\mathrm{norm}\;}\left(u,v\right)$. This normalization converts R(u,v) into a unit-magnitude spectrum, in which the magnitude of every frequency component is equal to 1, thereby preserving only the phase difference information:   

(11) $$R_{\mathrm{norm}\;}\left(u,v\right)=\frac{R(u,v)}{|R(u,v)|+\varepsilon }$$

where |R(u,v)| represents the magnitude of the cross-power spectrum, ϵ is a small positive constant introduced to prevent the denominator from becoming zero, and $R_{\mathrm{norm}\;}\left(u,v\right)$ represents the normalized cross-power spectrum. Then, the inverse Fourier transform is applied to $R_{\mathrm{norm}\;}\left(u,v\right)$ to convert it back to the spatial domain:

(12) $$r\left(x,y\right)=\left|F^{-1}\left\{R_{\mathrm{norm}\;}\left(u,v\right)\right\}\right|$$

where $F^{-1}\left\{*\right\}$ represents the inverse Fourier transform. In the resulting correlation map r(x,y), one or more correlation peaks are present, and the positions of the correlation peaks correspond to the displacements of different motion components. Since motion components with the same displacement contribute to the same correlation peak, the number of correlation peaks does not increase, while the response amplitude is enhanced. Therefore, the displacement information of the faint stellar background and space targets can be obtained by extracting several significant correlation peaks from r(x,y).

The estimation of target motion parameters is achieved by locating the correlation peak positions, which provide the displacement information of different motion components. To adaptively extract these motion components, potential local maxima in r(x,y) are first identified as candidate correlation peaks:

(13) $$\begin{array}{c} P=\left\{p|r\left(p\right)=\max_{q\in N_{\rho }\left(p\right)}r\left(q\right)\right\} \end{array}$$

where P denotes the candidate correlation peaks set, and $N_{\rho }\left(p\right)$ represents a square neighborhood centered at *p* with radius ρ=25. This operation is performed to suppress redundant responses around the same target. Since the correlation peaks with the highest amplitude does not necessarily indicate the most reliable motion estimate, the peak-to-sidelobe ratio (PSR) is introduced to evaluate the confidence of each correlation peak:

(14) $$\begin{array}{c} PSR\left(p\right)=\frac{r\left(p\right)-\mu_{S(p)}}{\sigma_{S(p)}+\varepsilon } \end{array}$$

where S(p) represents the sidelobe region surrounding the correlation peak with the main lobe excluded. The PSR quantitatively measures the prominence of the correlation peak relative to the surrounding sidelobes, with a higher PSR value indicating a more distinguishable correlation peak and a more reliable displacement estimation. To avoid directly combining quantities with different numerical scales, the raw correlation response $r_{i}\left(p\right)$ and the peak-to-sidelobe ratio $PSR_{i}\left(p\right)$ are first normalized by Min–Max normalization over the candidate peak set $P_{i}$ of the *i*-th correlation map and mapped into the interval [0,1]. The normalized values are denoted as ${\tilde{r}}_{i}\left(p\right)$ and ${\tilde{PSR}}_{i}\left(p\right)$, respectively. By jointly considering the correlation response amplitude and PSR, the ranking score $sco_{i}\left(p\right)$ is defined as

(15) $${\tilde{r}}_{i}\left(p\right)=\frac{r_{i}\left(p\right)-\min_{p\in P_{i}}r_{i}\left(p\right)}{\max_{p\in P_{i}}r_{i}\left(p\right)-\min_{p\in P_{i}}r_{i}\left(p\right)+\varepsilon }$$

(16) $${\tilde{PSR}}_{i}\left(p\right)=\frac{PSR_{i}\left(p\right)-\min_{p\in P_{i}}PSR_{i}\left(p\right)}{\max_{p\in P_{i}}PSR_{i}\left(p\right)-\min_{p\in P_{i}}PSR_{i}\left(p\right)+\varepsilon }$$

(17) $$\begin{array}{c} sco_{i}\left(p\right)={\tilde{r}}_{i}\left(p\right)\cdot {\tilde{PSR}}_{i}\left(p\right) \end{array}$$

For the *i*-th correlation map, the candidate peak with the highest ranking score is selected as the dominant background peak. Since the background stars share a consistent apparent motion, their motion responses are accumulated in the correlation map, resulting in the dominant correlation response peak. In contrast, space targets have independent relative motions, causing their corresponding correlation peaks to deviate from the background correlation peaks in the displacement space. Therefore, the target candidate correlation peak sequence PT is determined by removing candidate correlation peaks associated with the background motion, as follows:

(18) $$\begin{array}{c} p_{i}=\arg\max_{p\in P_{i}}sco_{i}\left(p\right) \end{array}$$

(19) $$\begin{array}{c} PB=\{p_{i}|i=1,\ldots ,n-1\} \end{array}$$

(20) $$\begin{array}{c} q_{i,k}\in \left\{p\in P_{i}\setminus \left\{p_{i}\right\}\;|\;{∥d\left(p\right)-d\left(p_{i}\right)∥}_{2}>\rho ,\;sco_{i}\left(p\right)\geq \tau_{s}\right\} \end{array}$$

(21) $$\begin{array}{c} PT=\{q_{i,k}|1\leq i\leq n-1,\;1\leq k\leq K_{i}\} \end{array}$$

where $p_{i}=\left(x_{i},y_{i}\right)$ denotes the displacement coordinates of the dominant background correlation peak in the *i*-th correlation map. $q_{i,k}=\left(x_{i,k},y_{i,k}\right)$ represents the coordinates of the *k*-th target candidate correlation peak in the *i*-th correlation map. These target candidate peaks are selected from the remaining significant peaks after excluding the neighbourhood of the background peak in displacement space, $\tau_{s}$ is an adaptive significance threshold computed from the normalized ranking scores in the *i*-th correlation map, and $K_{i}$ denotes the number of retained target candidate peaks in the *i*-th correlation map, which may vary among different correlation maps. Based on the displacement threshold $t_{d}$ (in pixels), similarity-based grouping is then performed on PB and PT to retain stable motion clusters with high inter-frame consistency:

(22) $$G\left\{\begin{array}{cc} G_{i} & {∥p_{i+1}-p_{i}∥}_{2}⩽t_{d}\\ G_{j} & \mathrm{otherwise}\\ \ldots & \end{array}\right.$$

(23) $$H\left\{\begin{array}{cc} H_{i} & {∥q_{i+1,k}-q_{i,k}∥}_{2}⩽t_{d}\\ H_{j} & \mathrm{otherwise}\\ \ldots & \end{array}\right.$$

(24) $$\begin{array}{cc} {∥p_{i+1}-P_{i}∥}_{2} & ={∥{\left(x,y\right)}_{i+1}-{\left(x,y\right)}_{i}∥}_{2}\\ \; & =\sqrt{{\left(x_{i+1}-x_{i}\right)}^{2}+{\left(y_{i+1}-y_{i}\right)}^{2}} \end{array}$$

(25) $$\begin{array}{cc} {∥q_{i+1,k}-q_{i,k}∥}_{2} & ={∥{\left(x,y\right)}_{i+1,k}-{\left(x,y\right)}_{i,k}∥}_{2}\\ \; & =\sqrt{{\left(x_{i+1,k}-x_{i,k}\right)}^{2}+{\left(y_{i+1,k}-y_{i,k}\right)}^{2}} \end{array}$$

where ${∥p_{i+1}-p_{i}∥}_{2}$ and ${∥q_{i+1}-q_{i}∥}_{2}$ denote the Euclidean distances between adjacent candidate displacement points in PB and PT, respectively. For points in $P_{B}$ or $P_{T}$, if the Euclidean distance between two candidate displacement points is smaller than the threshold $t_{d}$, they are classified into the same subset. For the background candidate set *G*, the subset with the largest number of candidate points is retained:

(26) $$G_{max}=\underset{G_{i}\in G}{\arg\max}\left|G_{i}\right|$$

for the target candidate sets *H*, subsets containing no fewer than five candidate displacement points are retained, and the mean displacement of all points within each retained subset is calculated:

(27) $$H_{t}=\{H_{i}\in H|\left|H_{i}\right|\geq 5\}$$

where $|G_{i}|$ and $|H_{i}|$ denote the cardinalities (numbers of elements) of the corresponding subsets. $G_{max}$ represents the dominant background motion subset, and $H_{t}$ represents the retained target motion subsets.

(28) $$\begin{array}{cc} \; & x_{mean}^{b},y_{mean}^{b}=mean\left\{G_{max}\right\}\\ & =mean\left\{x_{1}+x_{2}+\cdots +x_{g}\right\},mean\left\{y_{1}+y_{2}+\cdots +y_{g}\right\} \end{array}$$

(29) $$\begin{array}{cc} \; & \left[\begin{array}{c} {\left(x_{mean}^{t},y_{mean}^{t}\right)}_{1}\\ \ldots \\ {\left(x_{mean}^{t},y_{mean}^{t}\right)}_{l} \end{array}\right]=mean\left\{H_{t}\right\}\\ \; & =mean\left\{\begin{array}{c} h_{1}\\ \ldots \\ h_{l} \end{array}\right.\\ \; & =\left\{\begin{array}{c} {\left(mean\left\{x_{1}+x_{2}+\ldots \right\},mean\left\{y_{1}+y_{2}+\ldots \right\}\right)}_{1}\\ \ldots \\ {\left(mean\left\{x_{1}+x_{2}+\ldots \right\},mean\left\{y_{1}+y_{2}+\ldots \right\}\right)}_{l} \end{array}\right. \end{array}$$

where $\left(x_{mean}^{b},y_{mean}^{b}\right)$ represents the average displacement of the dim star along the *x* and *y* axes, and $\left(x_{mean}^{t},y_{mean}^{t}\right)$ represents the average displacement of the space targets on the *x* axis and *y* axis. *g* represents the number of points in $G_{max}$, and *l* represents the number of subsets in $H_{t}$. $\left\{h_{1},h_{2},\ldots ,h_{l}\right\}\in H_{t}$ represents the set of targets with a point count greater than or equal to 5. Thus, the motion parameters of the dim star background and the spatial targets can be estimated as follows:

(30) $$Dis^{b}=\sqrt{{\left(x_{mean}^{b}\right)}^{2}+{\left(y_{mean}^{b}\right)}^{2}}$$

(31) $$Deg^{b}=atan2\left(y_{mean}^{b},x_{mean}^{b}\right)$$

(32) $$Dis^{t}=\sqrt{{\left(x_{mean}^{t}\right)}^{2}+{\left(y_{mean}^{t}\right)}^{2}}$$

(33) $$Deg^{t}=atan2\left(y_{mean}^{t},x_{mean}^{t}\right)$$

where $\left(Deg^{b},Dis^{b}\right)$ represent the motion direction and distance parameters of the dim star background, and ${\left(Deg^{t},Dis^{t}\right)}_{1,\ldots ,l}$ represents the motion direction and distance parameters of all spatial targets.

#### 3.2.2. Image Difference Fusion Coarse Processing

**Figure 4:**
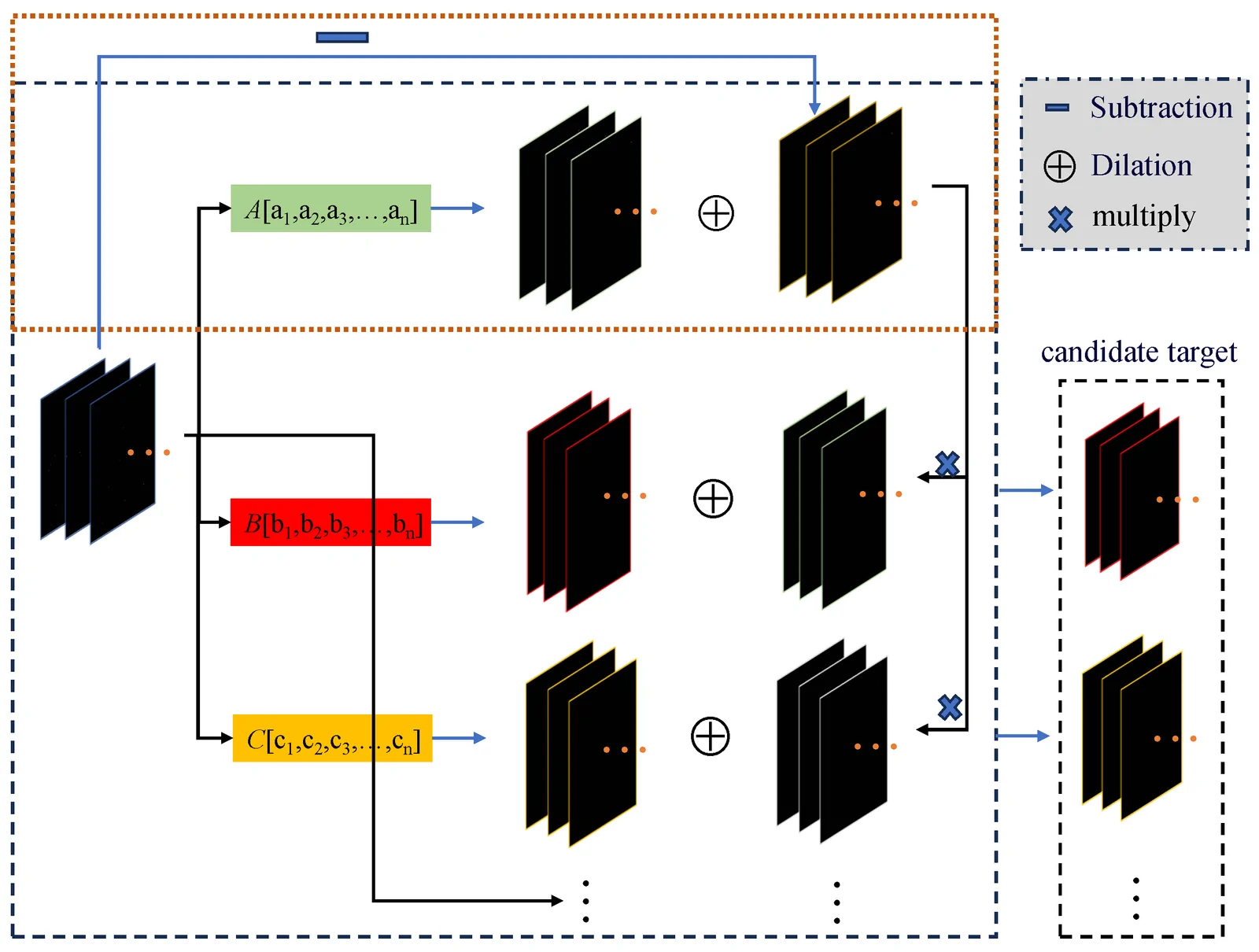
Image difference fusion coarse processing module (Arrows indicate the processing flow between different operations, and different colors represent different displacement components).

To distinguish between targets and faint stars and obtain candidate targets, this paper proposes an image difference fusion coarse processing module using estimated motion parameters. The detailed workflow of the image difference fusion coarse processing module is illustrated in Figure 4. Based on the differences in motion information between components, the image difference fusion technique can eliminate dim star interference while preserving target pixels.

First, adjust the displacement list based on the estimated motion parameters to obtain the reconstructed displacement sequence. For each point in $PB=\left\{p_{1},p_{2},\ldots ,p_{i},\ldots ,p_{n-1}\right\}$ and $PT=\{q_{i,k}|1\leq i\leq n-1,\;1\leq k\leq K_{i}\}$:

(34) $$p_{i}=\left\{\begin{array}{cc} {\left(x,y\right)}_{i} & {∥{\left(x,y\right)}_{i}-\left(x_{mean}^{b},y_{mean}^{b}\right)∥}_{2}\leq t_{d}\\ \left(x_{mean}^{b},y_{mean}^{b}\right) & \mathrm{otherwise} \end{array}\right.$$

(35) $$pf=\left\{p_{i}\right\}\left(i=1,2,\ldots ,n-1\right)$$

(36) $$q_{i,k}=\left\{\begin{array}{cc} {\left(x,y\right)}_{i,1} & {∥{\left(x,y\right)}_{i,1}-\left(x_{mean}^{t},y_{mean}^{t}\right)∥}_{2}\leq t_{d}\\ \vdots & \vdots \\ {\left(x,y\right)}_{i,K} & {∥{\left(x_{3},y_{3}\right)}_{i,K}-\left(x_{mean}^{t},y_{mean}^{t}\right)∥}_{2}\leq t_{d}\\ \left(x_{\mathrm{mean}}^{t},y_{\mathrm{mean}}^{t}\right) & \mathrm{otherwise} \end{array}\right.$$

(37) $$qf_{1,\ldots ,l}=\left\{q_{i,k}\right\}\left(i=1,2,\ldots ,n-1\right)$$

where pf and $qf_{1,\ldots ,l}$ represent the reconstructed displacement sequences of the faint stars and space targets, respectively.

Based on the differences in motion parameters between targets and faint stars, the image sequence is first displaced according to the faint star’s motion parameters, followed by a faint star region dilation operation. A differential operation is then performed between the subsequent frame and the dilated image, preserving target positions while eliminating residual faint stars, generating differential image sequences. In parallel, the image sequence undergoes a target region dilation operation after displacement based on target motion parameters to ensure spatial correspondence with subsequent frames, yielding candidate target image sequences. Finally, the candidate target sequence images are element-wise multiplied with the differential image sequences for further suppression of faint stars and enhancement of target signals. Based on the reconstructed displacement lists pf and $qf_{1,\ldots ,l}$, perform image difference fusion coarse processing on the sequence of images:

(38) $${\left(M_{B}\right)}_{i+1}=B_{i+1}-\left[{\left(B_{i}\right)}^{pf}⨁D_{1}\right]$$

(39) $${\left(M_{f}\right)}_{i+1}={\left(M_{B}\right)}_{i+1}\circ \left[B_{i+1}\circ \left({\left(B_{i}\right)}^{qf}⨁D_{2}\right)\right]$$

where $M_{f}$ represents the candidate target image sequences, $M_{B}$ is the differential image sequences, and ${\left(B\right)}^{pf}$ represents the pixel displacement operation of image sequence *B* according to the displacement list pf, with ${\left(B\right)}^{qf}$ following the same principle. $D_{1}$ is a 5×5 structural element, and $D_{2}$ is a 9×9 structural element. “⨁” represents the dilation operation, “−” represents the matrix difference operation, and “∘” represents matrix element-wise multiplication.

### 3.3. Directional Track Association

**Figure 5:**
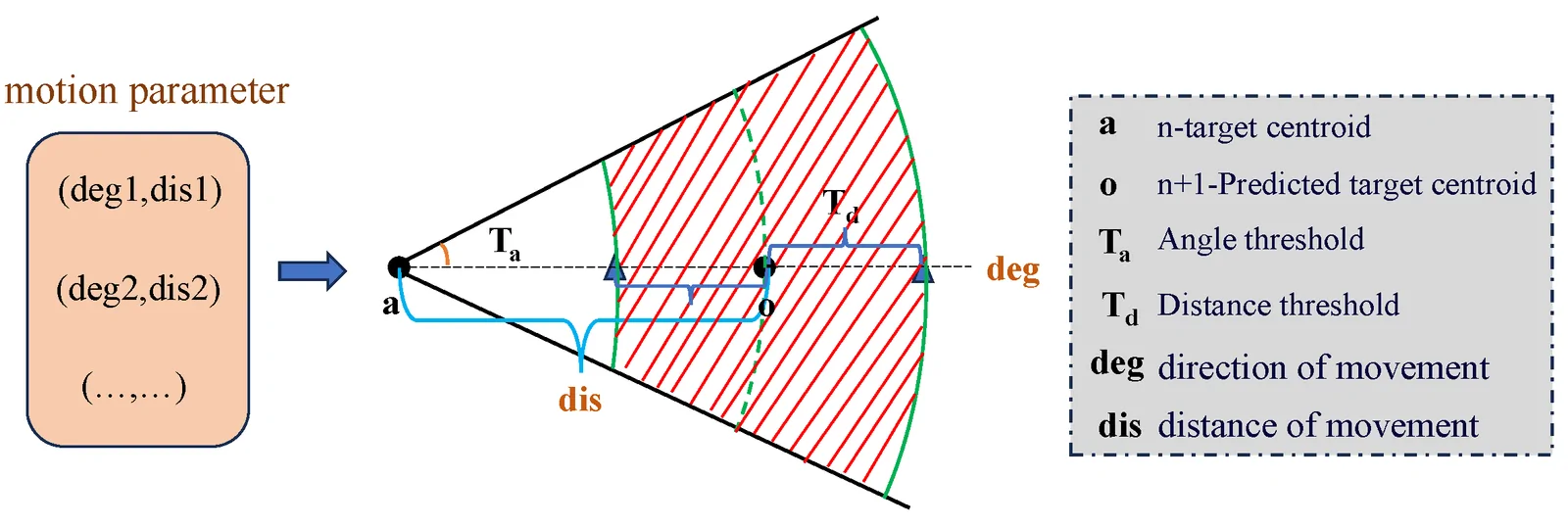
Directional track association diagram. (Arrows indicate the information flow and the red shaded sector represents the candidate search region constrained by motion parameters).

The candidate target image sequences $M_{f}$ contains real targets along with a small amount of clutter. To refine target detection, eliminate false alarms, and construct complete tracks, this paper designs a directional track association algorithm based on the estimated target motion parameters, candidate target image sequences, and differential image sequences (as shown in Figure 5). The displacement of targets across sequence frames exhibits regular continuity, generally following linear motion, while noise is highly random, with unpredictable positions and frame counts. This method can accurately guide target track association, narrowing the association range to a sector area, thereby effectively reducing the number of tracks and achieving precise detection and rapid extraction of real target track.

Using the candidate targets in $M_{f}$ and the target motion parameters to estimate the centroid position of the target in the next frame. Angle and distance thresholds are set as a criterion to delimit the predicted target range to a sector area, specifically the red area shown in Figure 5. Following this, track points that meet the conditions in the image frame are detected for track addition and recording, retaining motion-consistent target candidates while rejecting candidates that fail the motion validation criterion.

After obtaining the candidate target image sequences $M_{f}$, record all connected regions of the candidate targets and save their centroids:

(40) $$\cdots \to M_{f}^{i}\left\{\begin{array}{c} a\left(x_{a},y_{a}\right)\\ b\left(x_{b},y_{b}\right)\\ c\left(x_{c},y_{c}\right)\\ \ldots \end{array}\right.\to M_{f}^{i+1}\left\{\begin{array}{c} d\left(x_{d},y_{d}\right)\\ e\left(x_{e},y_{e}\right)\\ f\left(x_{f},y_{f}\right)\\ \ldots \end{array}\right.\to \ldots$$

where a,b,c,… represent all the connected regions in image $M_{f}^{i}$, and $\left(x_{a},y_{a}\right),\left(x_{b},y_{b}\right),\left(x_{c},y_{c}\right),\ldots$ represents the corresponding centroids of the connected regions. $M_{f}^{i+1}$ is the subsequent frame of image $M_{f}^{i}$, and $i=\left(1,2,\ldots ,n\right)$ denotes the frame index of the sequence.

A coarse motion gate is first applied to eliminate candidates with inconsistent displacement. An angle threshold $T_{a}=20^{{}^{\circ}}$ (in degrees) and a distance threshold $T_{d}=30$ (in pixels) are set. It should be noted that the angular threshold $T_{a}$ and distance threshold $T_{d}$ are not used as velocity-independent global search thresholds. In the proposed association process, the target position in frame i+1 is first predicted from the centroid in frame *i* and the estimated motion parameters. The thresholds $T_{a}$ and $T_{d}$ are then applied only as a local residual-tolerance gate around the predicted position, so as to tolerate small prediction errors and avoid missed associations. Pair the connected regions from adjacent frames, and determine whether they form a track based on the motion parameters and thresholds, i.e., whether they conform to the target motion state. For example, consider track ad as a case:

(41) $$\vec{ad}\left\{\begin{array}{c} Dis^{t}-T_{d}\leq \sqrt{{\left(x_{d}-x_{a}\right)}^{2}+{\left(y_{d}-y_{a}\right)}^{2}}\leq Dis^{t}+T_{d}\\ Deg^{t}-T_{a}\leq atan2\left(y_{d}-y_{a},x_{d}-x_{a}\right)\leq Deg^{t}+T_{a} \end{array}\right.$$

candidate points satisfying the predicted displacement distance and direction constraints are retained as potential associations. When multiple candidates satisfy the validation gate, the candidate with the minimum association cost is selected as the corresponding trajectory association point.

(42) $$z^{*}=\arg\min_{z\in G}J\left(z\right)$$

where $z^{*}$ denotes the finally selected trajectory association point, *G* represents the set of candidate points satisfying the motion-based validation gate and *J* denotes the association cost, which is defined as

(43) $$J={∥z-o∥}_{2}$$

where z∈G denotes a candidate point within the validation gate, *o* represents the predicted position based on the estimated motion parameters, and *J* denotes the association cost defined as the Euclidean distance between the candidate point *z* and the predicted position *o*. Finally, check whether all generated tracks $\vec{traj}$ are real tracks:

(44) $$\left\{\begin{array}{ccc} \vec{traj} & \geq 5, & \mathrm{valid}\;\mathrm{trajectory},\\ \vec{traj} & <5, & \mathrm{false}\;\mathrm{trajectory}. \end{array}\right.$$

A trajectory is regarded as a valid target trajectory if the number of successfully associated frames is greater than or equal to five. This criterion serves as a trajectory confirmation rule, rather than a strict continuous detection requirement. Although random false alarms may occasionally satisfy the motion validation gate in a few frames, they are unlikely to form a motion-consistent trajectory over multiple frames. The threshold of five associated frames provides a balance between rejecting short false tracks and retaining dim targets with intermittent visibility. Short-term missed detections are allowed and recovered using the trajectory completion strategy. For the trajectories judged to be true, this paper will further examine the issue of discontinuity in inter-frame target tracks. For trajectories with intermittent missing detections, the proposed directional track association is further applied to the corresponding differential image sequences $M_{B}$ to recover missing positions and generate complete trajectories. The result is a sequence of images $M_{w}$ and a multi-frame trajectory record file *F*. The overall process of the algorithm is described in Algorithm 1.

In this procedure, the outputs of each stage are used as constraints for the following stage. Errors from earlier stages may propagate to subsequent modules. Registration residuals may leave stellar structures in the processed images, which can introduce additional responses in the phase-correlation map and generate false candidate peaks. To reduce this influence, connected domain post-processing is applied after bright star removal, while local non-maximum suppression and PSR-based peak ranking are used in the phase estimation stage to suppress unstable responses. The reconstructed displacement sequences further reject abnormal single-frame estimates through inter-frame consistency screening. In the image difference fusion stage, dilation operations provide tolerance to small residual registration and displacement-estimation errors. Finally, the directional track association module uses both distance and direction constraints to remove motion-inconsistent candidates. Therefore, the influence of registration and phase-estimation errors is progressively constrained by candidate filtering, displacement reconstruction, image difference fusion, and trajectory validation.
**Algorithm 1** Proposed Algorithm**Require:** Adjacent frames image $I_{i}$ and $I_{i+1}$.
**Ensure:** Result images $M_{w}$, trajectory recording file *F*.
  1:Perform bright star removal and connected domain post-processing to obtain the processed images $P_{i}$ and $B_{i}$, respectively.  2:Generate the inter-frame phase difference spectral correlation image $r\left(x,y\right)$, search for prominent correlation peaks in $r\left(x,y\right)$ and record them in sets PB and PT.  3:**for** $PB=p_{1}:\left(p_{n-1}\right)$, $PT=q_{1}:\left(q_{n-1}\right)$ **do**  4:     Similarity judgment ${∥p_{i+1}-p_{i}∥}_{2}⩽t_{d}$, ${∥q_{i+1,k}-q_{i,k}∥}_{2}⩽t_{d}$, obtain similarity sets *G* and *H*. Find the set in *G* with the most points $G_{max}$, and the sets in *H* with more than 5 points $H_{t}$.  5:     **for** $G_{max},\left(h_{1}:h_{l}\right)\in H_{t}$ **do**  6:         Calculate average displacement $\left(x_{mean}^{b},y_{mean}^{b}\right)$ and ${\left(x_{mean}^{t},y_{mean}^{t}\right)}_{1,\ldots ,l}$; obtain motion parameters $\left(Deg^{b},Dis^{b}\right)$, ${\left(Deg^{t},Dis^{t}\right)}_{1,\ldots ,l}$ and reconstructed displacement lists pf, $qf_{1,\ldots l}$.  7:     **end for**  8:**end for**  9:Perform image differential fusion coarse processing, obtain candidate target image sequences $M_{f}$ and differential image sequences $M_{B}$.10:Detect all connected domains in $M_{f}^{i}$ and $M_{f}^{i+1}$, denoted as $\left\{a,b,c,\ldots \right\}$ and $\left\{d,e,f,\ldots \right\}$ respectively, and save their centroid coordinates.11:**for** $\left\{a,b,c,\ldots \right\}$, $\left\{d,e,f,\ldots \right\}$ **do**12:    Determine whether any two connected domains (e.g., $\vec{ad}$) in adjacent frames form a trajectory, based on the target motion parameters $Dis^{t}$, $Deg^{t}$ and thresholds $T_{a}$, $T_{d}$.13:    **for** Each $\vec{traj}$ **do**14:          **if** $\vec{traj}⩾5$ **then**15:             Record all target connected domains and their centroid coordinates on the trajectory. For confirmed trajectories with missing detections, predict the missing-frame position using the estimated motion parameters and the nearest valid trajectory point. Search connected components in the corresponding differential image $M_{B}^{i}$ within the motion validation gate defined by $T_{a}$ and $T_{d}$. If multiple candidates satisfy the gate, select the candidate with the minimum Euclidean distance to the predicted position and update $M_{w}$ and *F*. The final output consists of an image sequence $M_{w}$ and a multi-frame trajectory record file *F*.16:          **else**17:                Delete this trajectory.18:          **end if**19:    **end for**20:**end for**

## 4. Experiments and Discussion

To validate the effectiveness of the proposed method, experiments were conducted on four star map sequences. The experimental results were analyzed from both qualitative and quantitative perspectives. In addition, the proposed method was compared with several state-of-the-art methods, including infrared moving small-target detection using STLDM [24], infrared dim and small target detection in complex scenes via MFSTPT [25], clustering and tracking-guided infrared spatial–temporal small target detection (CTSTC) [26], the multiframe spatio-temporal attention motion-adaptive network (MSAMNet) [30], and the temporal evolution-aware network with spatial guidance (TESNet) [31]. All experiments were conducted on a PC equipped with an Intel Core i5-6300HQ CPU (Intel Corporation, Santa Clara, CA, USA) and 4 GB RAM. The experiments were implemented using Python 3.9.20, and Visual Studio Code 1.82.0 (Microsoft Corporation, Redmond, WA, USA) was used as the development environment.

### 4.1. Datasets Introduction

We conducted tests on four star map sequences, namely sequence 1, sequence 2, sequence 3 and sequence 4, which contain 51, 26, 50 and 50 images, respectively. Detailed descriptions of the datasets are given in Table 1. The selected datasets cover different sky regions, and thus can effectively evaluate the performance of the proposed method.

The image sequences were acquired in Xinglong County, Hebei Province, China, using a self-developed ground-based optical observation camera. The camera adopts a CMOS image sensor as the core imaging device and is mounted on an altitude-azimuth telescope mount to achieve accurate pointing and tracking during celestial observations. The observations were conducted on 17 June and 18 June 2024. These real-world observation sequences were collected under dense stellar backgrounds and contain dim moving space targets, providing realistic scenarios for evaluating the proposed detection and track association method.

The image sequences used in this study are observation sequences represented in the stellar background reference frame. The ground truth of each sequence consists of manually annotated binary target masks and the corresponding target centroids. The binary masks were generated by labeling the visible target regions in each annotated frame, and the centroid of each target was then calculated from the labeled mask. The centroid annotations are stored as (x,y) coordinates with frame indices. For frames containing multiple targets or multiple target segments, each target instance was labeled independently. In addition, the exposure times of the four image sequences are 10 s, 20 s, 150 ms, and 300 ms, respectively.

### 4.2. Evaluation Metrics

In order to quantitatively assess the overall performance of the proposed algorithm, multiple evaluation metrics were used, including precision (Pre), recall (Re), false alarm rate (Fa), receiver operating characteristic (ROC) curve, precision–recall (PR) curve, and area under curve (AUC). Considering the extremely sparse distribution of dim space targets in high-resolution stellar images, different statistical levels are adopted for target detection accuracy and background false alarm evaluation. Specifically, Pre and Re are calculated at the object level, while $F_{a}$ is calculated at the pixel level. For object-level evaluation, a detected connected component is regarded as a true positive object ($TP_{obj}$) if its centroid is located within a predefined distance threshold from the ground-truth target position. Otherwise, it is counted as a false positive object ($FP_{obj}$). A ground-truth target without a matched detection is regarded as a false negative object ($FN_{obj}$). Therefore, Pre and Re are defined as

(45) $$Pre=\frac{TP_{obj}}{TP_{obj}+FP_{obj}}$$

(46) $$Re=\frac{TP_{obj}}{TP_{obj}+FN_{obj}}$$

precision measures the proportion of correctly detected targets among all detected objects, while recall measures the proportion of successfully detected targets among all ground-truth targets.

To evaluate background suppression capability, the false alarm rate is calculated at the pixel level:

(47) $$F_{a}=\frac{FP_{pix}}{FP_{pix}+TN_{pix}}$$

where $FP_{pix}$ represents the number of background pixels incorrectly classified as target pixels, and $TN_{pix}$ represents the number of correctly classified background pixels.

The ROC curve describes the relationship between the object-level true positive rate (TPR) and the pixel-level false positive rate (FPR) under different thresholds. Here, TPR is equivalent to Re, and FPR corresponds to $F_{a}$. The PR curve represents the relationship between object-level precision and recall under different thresholds. The PR–AUC is calculated from the PR curve to evaluate the overall target detection performance. Since dim space target detection requires operation under extremely low false alarm conditions, the ROC performance is evaluated using the partial area under the ROC curve ($pAUC_{ROC}$) within the low false alarm region ($F_{a}\leq 10^{-5}$), instead of the full-range ROC–AUC from 0 to 1.

The signal-to-noise ratio (SNR) can describe the degree of contrast between the target area and its surroundings, which is defined as follows:

(48) $$SNR=\frac{\left|\mu_{t}-\mu_{b}\right|}{\sigma_{b}}$$

where $\mu_{t}$ denotes the average pixel value of the target, and $\sigma_{b}$ and $\mu_{b}$ represents the standard deviation and the average value of pixels in the neighboring region.

We also employ the intersection over union (IoU) to evaluate the proposed method. IoU is a pixel-level metric that describes the accuracy of the model by calculating the overlap between the predicted region and the ground truth region, which is defined as follows:

(49) $$IoU=\frac{TP}{T+P-TP}$$

where TP, *T* and *P* denote the true positive, true and positive, respectively.

In addition, the deviation error between the predicted trajectory centroid and the actual centroid is measured using the following metric:

(50) $$d=\sqrt{\frac{1}{m}\sum_{i=1}^{m}\left[{\left(x_{i}^{\prime }-x_{i}\right)}^{2}+{\left(y_{i}^{\prime }-y_{i}\right)}^{2}\right]}$$

where $\left(x_{i}^{\prime },y_{i}^{\prime }\right)$ is the predicted centroid of the *i*-th target, $\left(x_{i},y_{i}\right)$ is the actual centroid of the *i*-th target, and *m* is the number of targets.

### 4.3. Implementation Details of Compared Methods

To ensure the fairness and reproducibility of the comparison experiments, all compared methods were implemented using the parameter configurations recommended in their original papers. For traditional model-based methods, including STLDM, CTSTC, and MFSTPT, the key parameters were kept consistent with the original settings. For learning-based methods, including MSAMNet and TESNet, the original network architectures and training strategies were adopted. The detailed parameter settings of different comparison methods are listed in Table 2.

For learning-based methods, including MSAMNet and TESNet, the original network architectures were retained and the recommended training strategies were followed. To avoid data leakage, the four evaluated sequences used in this paper were strictly excluded from the fine-tuning process and were used only for testing. The models were fine-tuned for 100 epochs using the BUAA-MSOD dataset introduced in the MSAMNet study, which contains additional space-target samples with similar imaging characteristics but does not overlap with the four test sequences used in this work. No frames, target annotations, or temporally adjacent image patches from the evaluated sequences were used during training, validation, hyperparameter selection, or fine-tuning.

### 4.4. Qualitative Comparisons

**Figure 6:**
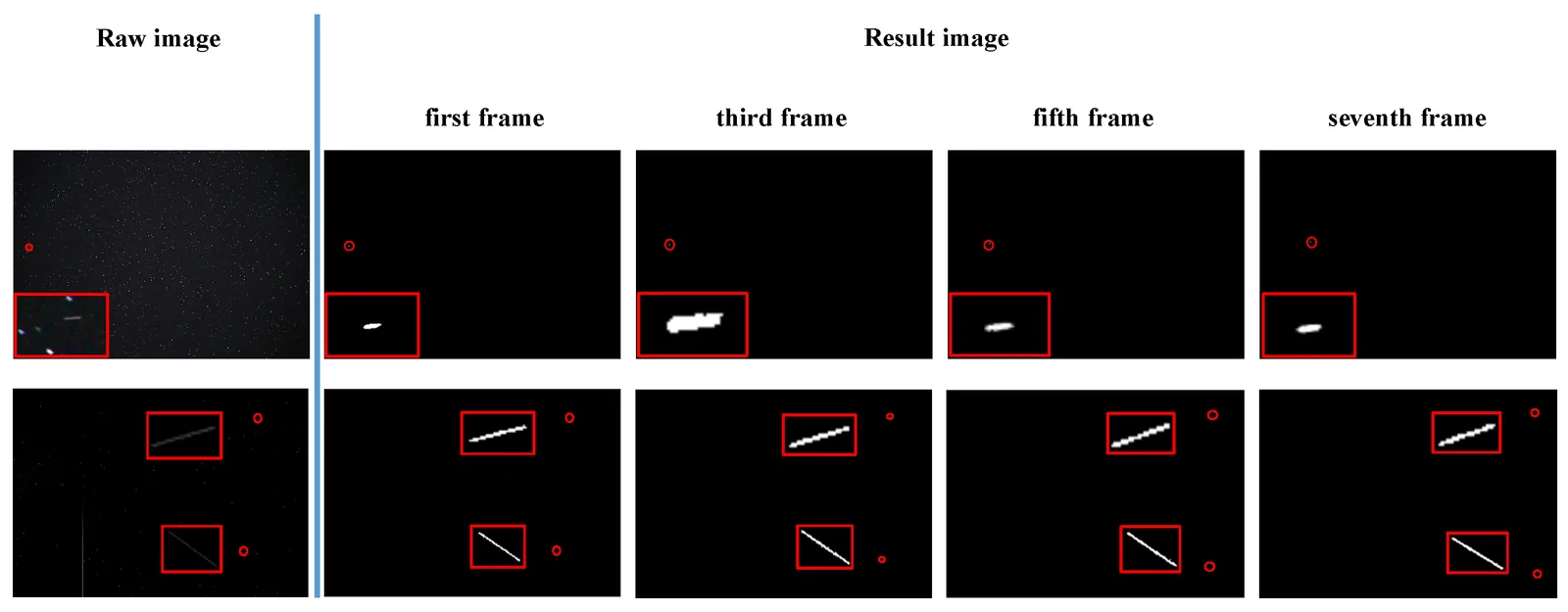
Space target detection result example diagram of our method. Red circles indicate target locations, and red boxes denote zoom-in regions for detailed visualization.

**Figure 7:**
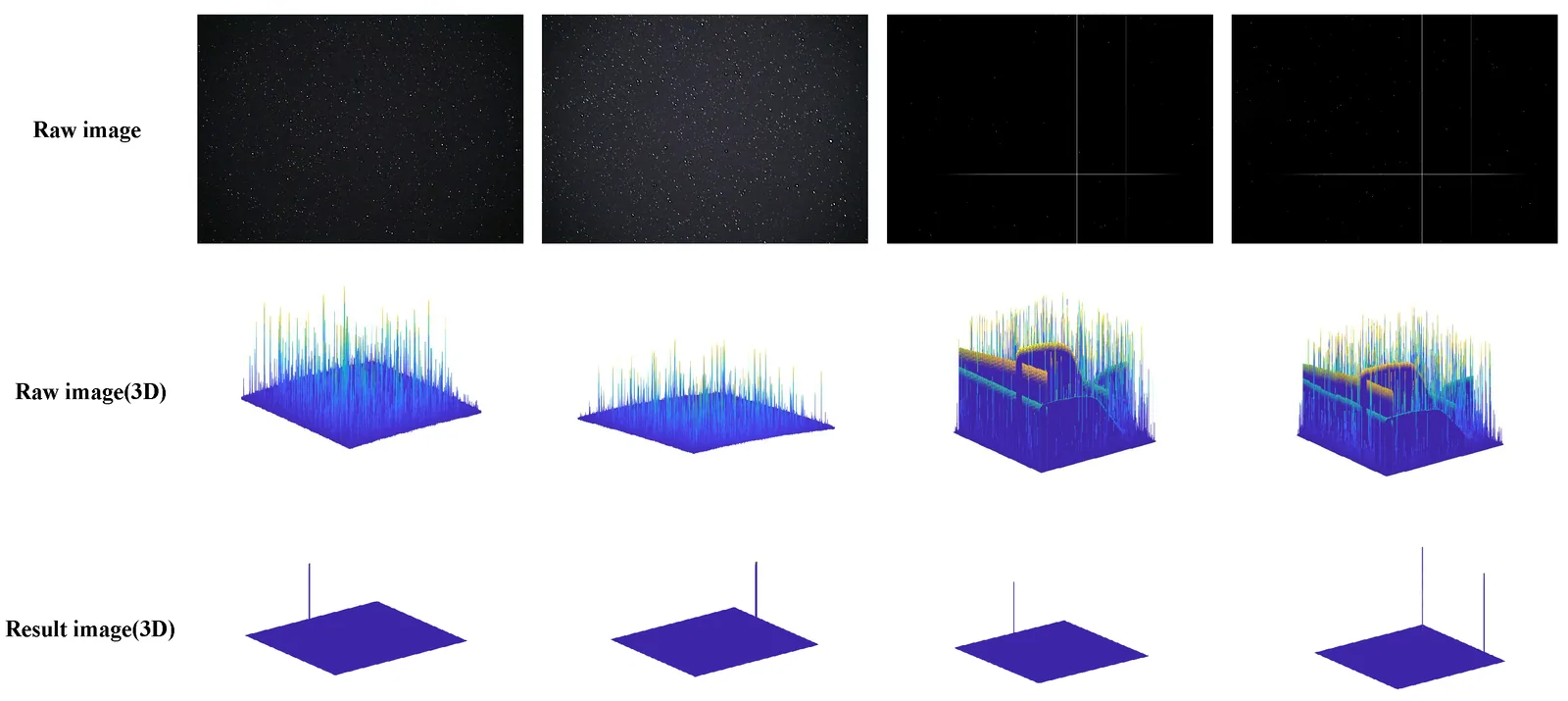
Stellar background suppression demonstration diagram of our method (the first row shows the original images, the second row is a 3D display of the original images, and the third row presents the corresponding 3D visualization of the detection results).

To validate the detection capability of the proposed algorithm, experiments were conducted on multiple spatial sequence images. The results demonstrated effective detection of faint spatial targets and precise suppression of dense star backgrounds, as illustrated in Figure 6 and Figure 7.

In Figure 6, the first column displays representative frames of two original image sequences, showing three dim and faint space targets with distinct motion states. The second column shows the corresponding partial detection result image frames, and it can be observed that our method significantly enhances the shape and brightness of spatial targets, achieving robust detection for targets of varying magnitudes and motion states.

In Figure 7, the first row is the original image frame, the second row is the 3D display corresponding to the original image, and the third row is the 3D display corresponding to the detection result. It can be seen that our method can effectively suppress the interference of stars with different brightnesses, and then detect the real target with good background suppression.

**Figure 8:**
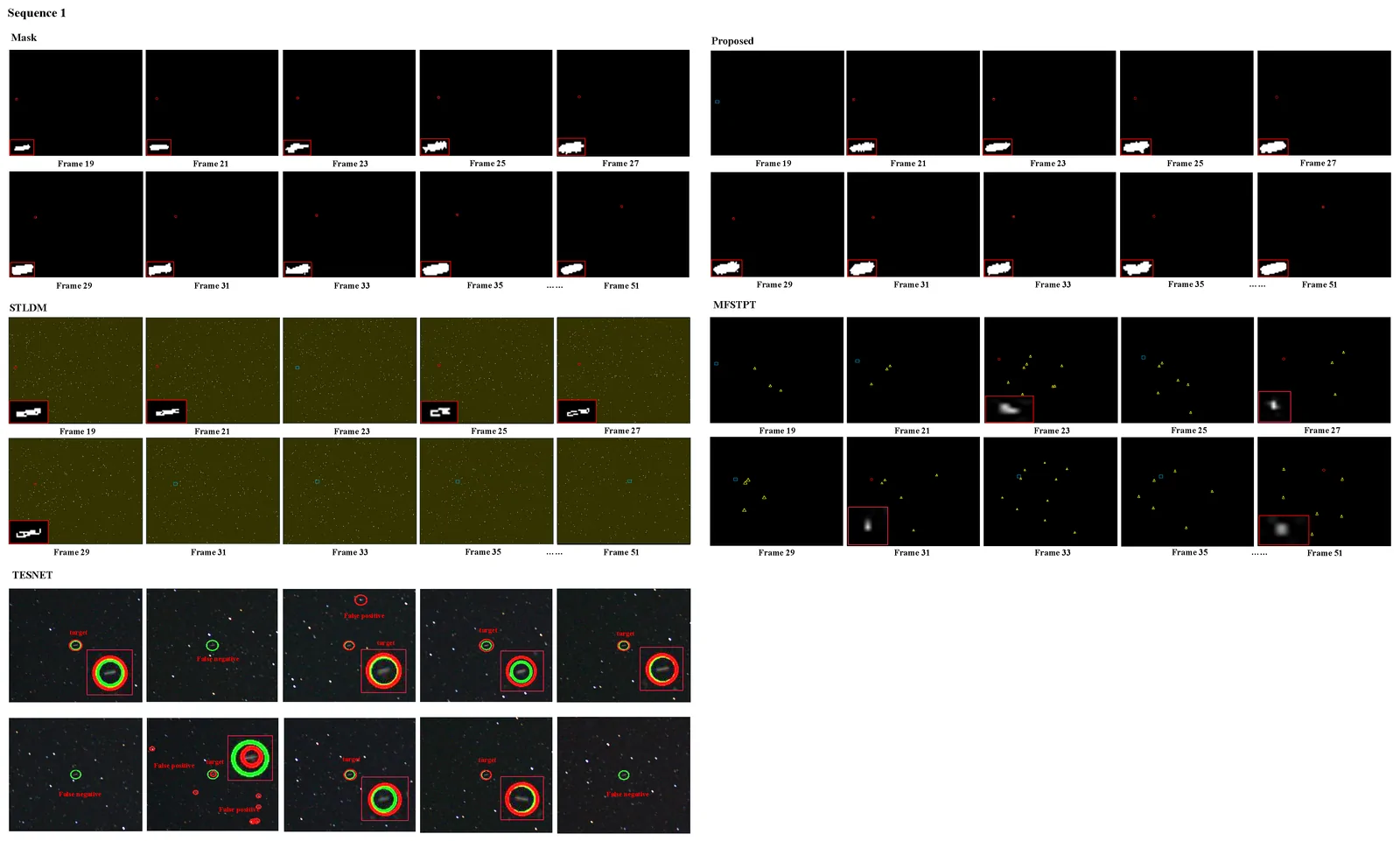
Comparison of detection results on several frames of sequence 1 among different methods.

**Figure 9:**
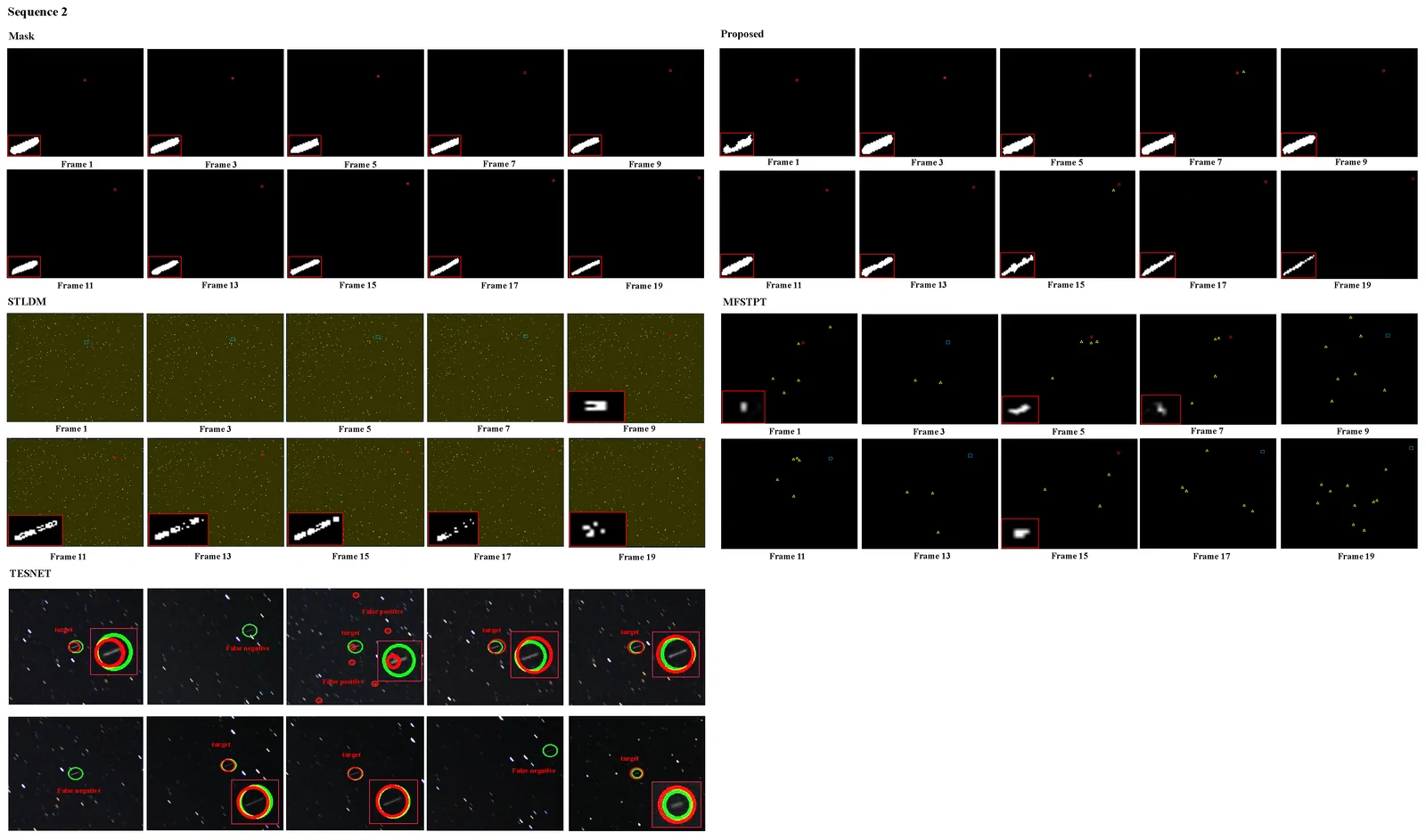
Comparison of detection results on several frames of sequence2 among different methods.

**Figure 10:**
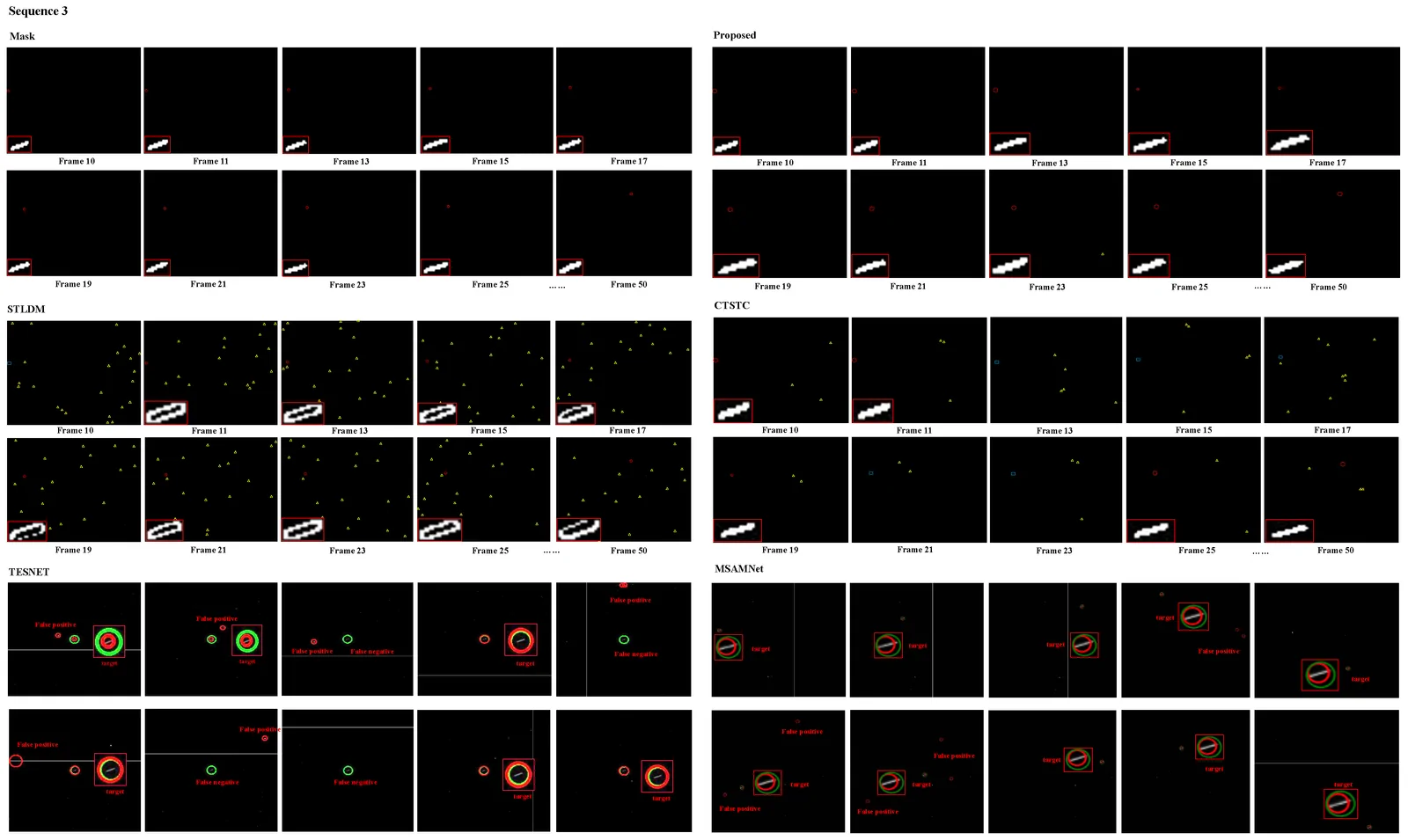
Comparison of detection results on several frames of sequence3 among different methods.

**Figure 11:**
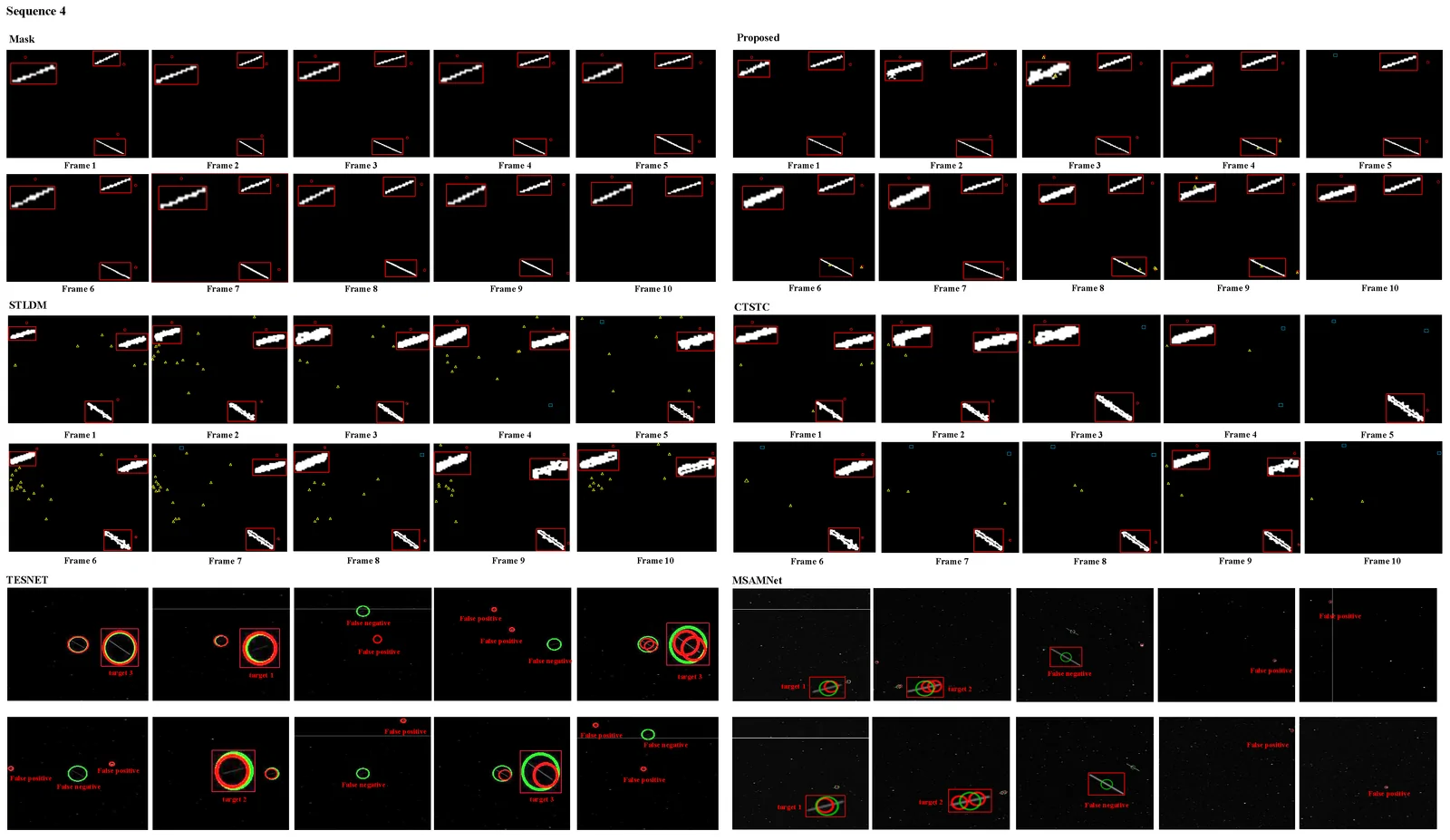
Comparison of detection results on several frames of sequence4 among different methods.

In order to better validate the detection performance of the proposed algorithm, this study compares four state-of-the-art detection methods. We conducted comparative experiments of each method on four datasets, and selected some frames from the detection results to be shown as visualization results, as shown in Figure 8, Figure 9, Figure 10 and Figure 11. Figure 8, Figure 9, Figure 10 and Figure 11 show partial detection results of different methods on sequences 1–4, respectively. Red circles denote ground-truth targets, blue boxes indicate missed detections, and yellow triangles mark false alarms. When the number of false alarms is excessively large, the entire image is overlaid with a yellow region for visualization. It should be noted that MSAMNet and TESNet adopt a different visualization scheme: green circles denote ground-truth targets, while red circles represent detection results. The coexistence of green and red circles indicates correct target detection, a red circle alone signifies a false alarm, whereas a green circle alone corresponds to a missed detection. The magnified view of the target region is displayed near the target. In these sequence images, the targets exhibit diverse motion directions and velocities, while dense background stars of varying brightness impose significant interference. Additionally, the low SNR of the images causes spatial targets to be submerged in the stellar background, further complicating detection. Experimental results demonstrate that the proposed method achieves robust detection performance for faint spatial targets under dense stellar backgrounds.

From the comparative results, it can be observed that STLDM, which detects targets based on local contrast differences, struggles to effectively distinguish stars from spatial targets with similar characteristics. As a result, it produces a large number of false alarms and exhibits insufficient suppression of the stellar background. The MFSTPT method, grounded in the low-rank and sparse assumption, achieves better background suppression. However, the dense stars surrounding spatial targets violate the sparse assumption, causing the targets to be filtered out as noise and resulting in a higher rate of missed detections, thereby degrading spatial target detection performance. CTSTC incorporates Bayesian inference-based tracking regularization to model target motion and state transitions, thereby improving robustness against interference while maintaining high detection accuracy with fewer false alarms. MSAMNet enhances the representation capability of joint spatio-temporal features, achieving higher detection accuracy and a lower false alarm rate. However, in complex backgrounds with densely distributed bright stars, it still struggles to effectively detect dim targets submerged in the stellar background. TESNet introduces a lightweight 3D convolutional backbone, a dual-branch spatio-temporal feature extraction structure, and other modules to achieve adaptive fusion of spatial and temporal information, thereby further improving the robustness of dim and small space target detection in various deep-space observation scenarios. Since MSAMNet and CTSTC cannot detect any targets in sequence 1 and sequence 2, and MFSTPT achieves no detection results in sequence 3 and sequence 4, corresponding quantitative statistics are not presented. Experimental results demonstrate that the proposed method effectively leverages the distribution characteristics and temporal motion information of spatial targets. It can accurately suppress background interference caused by stars with varying brightness levels while enhancing their robust detection capabilities for spatial targets of different magnitudes. Therefore, under dense stellar backgrounds, the proposed method achieves excellent detection performance for faint spatial targets with diverse motion parameters.

### 4.5. Quantitative Comparisons

As shown in Table 3, quantitative evaluations of all methods are conducted on four datasets using the metrics Pre, Re, and Fa. Due to the large number of false alarms, STLDM exhibits notably low Pre across all datasets. MFSTPT and CTSTC suffer from frequent missed detections, resulting in poor overall performance in terms of both Pre and Re. By integrating spatiotemporal modeling with an adaptive attention mechanism, MSAMNet improves detection accuracy and reduces the false alarm rate to some extent. However, it is still prone to false detections or missed detections in dim target detection scenarios. TESNet demonstrates strong robustness under complex stellar clutter and low signal-to-noise ratio conditions, achieving a relatively balanced performance in Pre and Re across all datasets. In contrast, the proposed method achieves the best performance on all four datasets, significantly outperforming other methods in terms of Pre and Re while maintaining a lower Fa. The experimental results indicate that missed detections may occur only when the motion state of a spatial target in a certain frame deviates significantly from its average motion pattern. In addition, when residual stellar interference exists near a spatial target, directional trajectory detection may misclassify such noise as a valid target, resulting in a small number of false alarms.

For the failed cases reported in the comparison experiments, we further examined the output results of the competing methods. The failures mainly occurred when excessive stellar residuals, low target contrast, or fragmented network responses prevented the methods from producing valid target trajectories. For traditional methods, dense stellar interference often generated a large number of false alarms that overwhelmed the real target responses. For learning-based methods, the predicted target responses were sometimes too weak after thresholding or too fragmented to form valid connected components under the same object-level matching criterion. Therefore, “Failed” indicates that no valid target trajectory satisfying the evaluation criterion was obtained, rather than that the code failed to execute.

**Figure 12:**
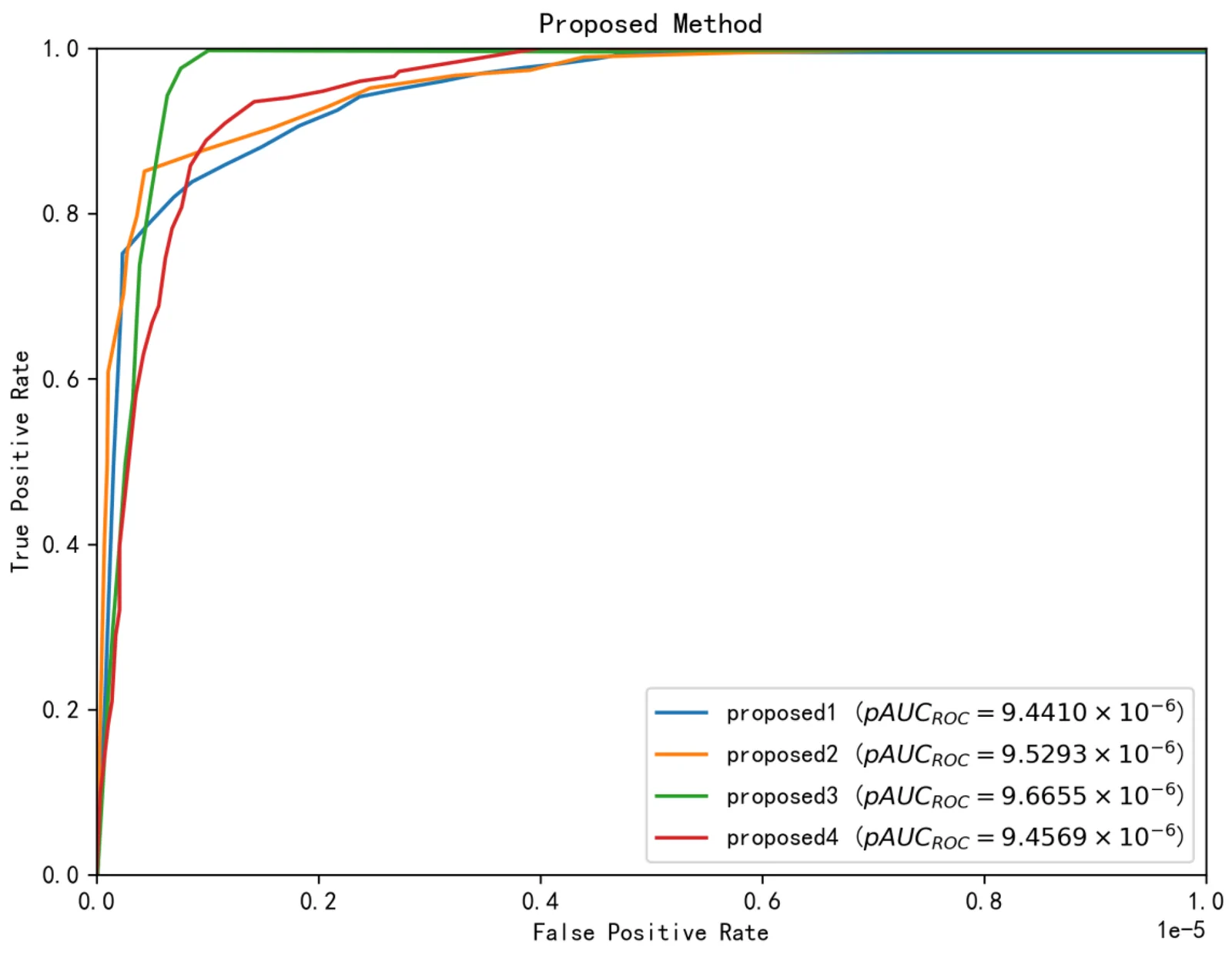
ROC curves of the proposed method on four datasets.

**Figure 13:**
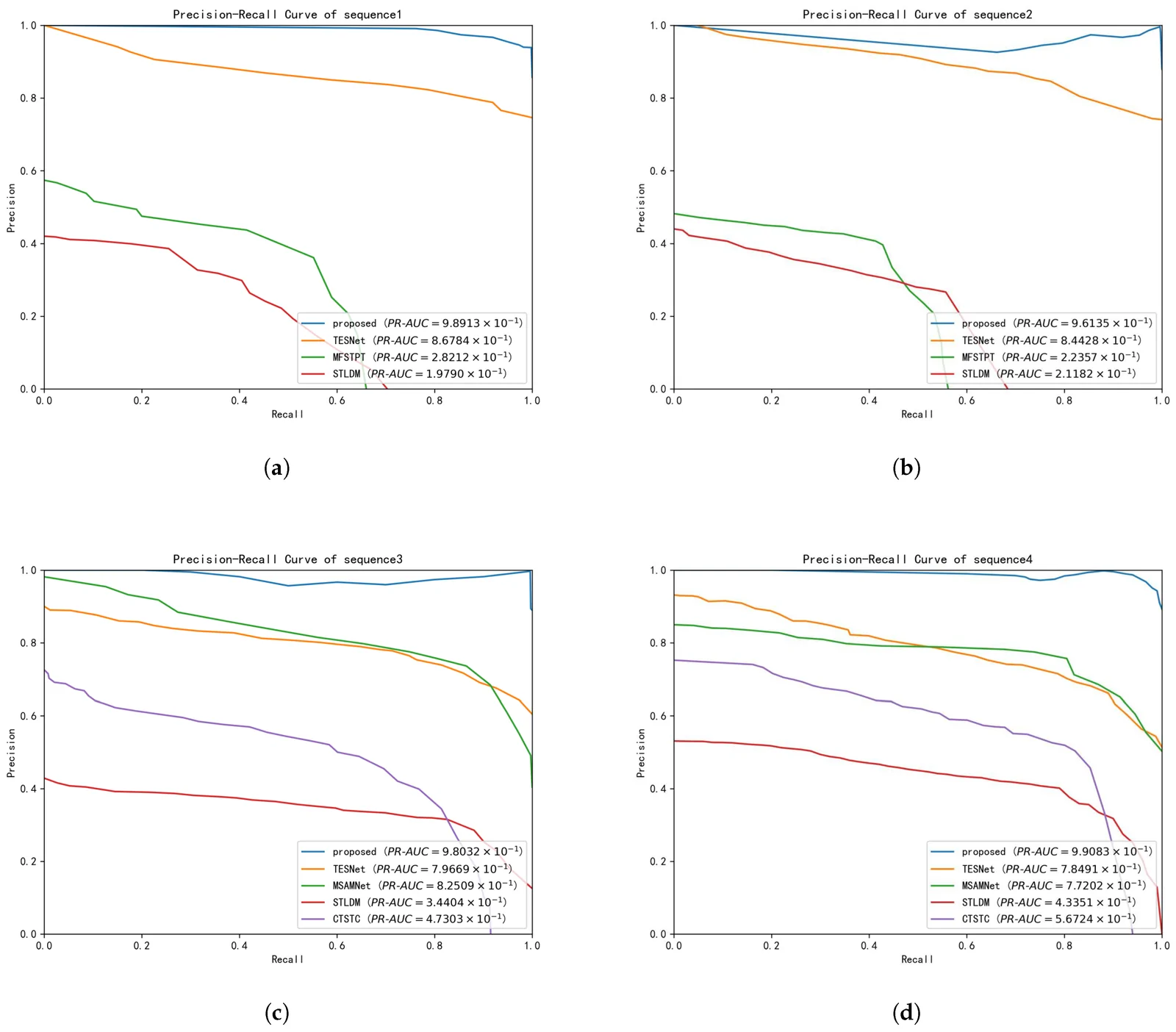
PR curves achieved by different methods on four datasets: (**a**) sequence 1, (**b**) sequence 2, (**c**) sequence 3, (**d**) sequence 4.

The ROC curves of the proposed method on the four datasets are shown in Figure 12, while the PR curves of different comparative methods on the four datasets are presented in Figure 13.

As shown in Figure 12 and Table 4, the proposed method achieves the best $pROC_{AUC}$ performance on all four datasets with SNR of 2.66, 1.39, 0.72, and 0.65, yielding values of $9.44\times 10^{-6}$, $9.53\times 10^{-6}$, $9.67\times 10^{-6}$, and $9.46\times 10^{-6}$, respectively. As shown in Figure 13 and Table 4, despite the gradual decrease in SNR, the proposed method still maintains high precision and recall, demonstrating stable and robust PR-AUC performance, with values of 0.989, 0.961, 0.980, and 0.991, respectively.

Table 5 gives the IoU results obtained by various methods. In this work, IoU is calculated over all annotated frames to jointly evaluate the target detection completeness and segmentation accuracy. If the target is missed and no predicted region is obtained, the IoU of this frame is assigned as zero. As demonstrated, the proposed method maintains robust detection and segmentation performance across varying SNR, achieving IoU values of 0.672, 0.718, 0.885, and 0.748 on the four datasets, respectively. It preserves the original shape structure of detected targets while ensuring high detection accuracy.

Table 6 presents the average positional deviation errors (in pixels) of various methods across the four datasets. Since sequence 4 contains three dim space targets, the corresponding results are reported separately as target1, target2 and target3. The proposed method achieves the lowest localization error on most datasets, with localization errors of 0.81, 2.57, 0.26, 1.22, 1.06 and 2.05 pixels for targets in each sequence, yielding an average error of only 1.33 pixels. MSAMNet and TESNet also exhibit favorable localization accuracy on some datasets. Specifically, MSAMNet achieves the best localization result on sequence 3, with a localization error of 0.18 pixels and an overall average error of 1.56 pixels. TESNet obtains the best localization result on sequence 1, with a localization error of 0.76 pixels and an overall average error of 1.51 pixels. In contrast, STLDM, CTSTC, and MFSTPT show average errors of 2.91, 2.29, and 3.06 pixels, respectively, which are significantly higher than those of the proposed method, indicating their relatively lower localization accuracy.

This paper further evaluates the computational efficiency of all algorithms on four sequence datasets, and the corresponding results are summarized in Table 7. The experimental results show that TESNet achieves the fastest running speed on sequence 1 and sequence 2, while MSAMNet obtains the optimal computational efficiency on sequence 3 and sequence 4. Both methods outperform the proposed method in terms of computational performance on the corresponding datasets. Nevertheless, compared with the other comparative algorithms, the proposed method still maintains favorable computational efficiency. In future work, the running time can be further reduced and the overall computational performance can be improved by optimizing the motion parameter estimation module.

To further analyze the computational cost of the proposed multi-stage framework, we decomposed the runtime into four major stages: bright star removal and connected domain processing (BSR-CDP), spectrum phase shift screening reconstruction (SPSR), image difference fusion coarse processing (IDFCP), and directional track association (DTA). For each dataset, ten consecutive frames containing valid target trajectories were selected, and the average per-frame runtime of each stage was measured. As shown in Table 8, SPSR is the dominant computational bottleneck across all datasets.

The total runtime in Table 8 is slightly higher than the full-sequence average runtime reported in Table 7 because Table 8 focuses on representative frames containing valid target trajectories, where the directional association and result generation steps are fully activated. In contrast, Table 7 reports the average runtime over the entire sequence.

It should be noted that the runtime comparison is affected by hardware configuration and implementation strategy. The proposed method and traditional model-based methods were implemented as CPU-based sequential algorithms without GPU acceleration or batch processing. In contrast, learning-based methods such as MSAMNet and TESNet performed model inference on GPU and used batch inference during testing, while image reading, preprocessing, postprocessing, and metric calculation were still mainly conducted on CPU. Therefore, the runtime of learning-based methods reflects both GPU inference and CPU-side data processing.

In terms of scalability, the main computational cost of the proposed method comes from the spectrum phase shift screening and reconstruction stage, where FFT-based phase correlation is performed between adjacent frames. For an image of size H×W, this stage has an approximate complexity of O(HWlog(HW)), while the other stages are mainly pixel-wise or candidate-wise operations. The memory usage of the proposed method scales approximately with the image size, i.e., O(HW), because only several image-sized arrays and Fourier-domain arrays are required for adjacent-frame processing. For larger images or higher-frame-rate sequences, GPU-based FFT acceleration, image tiling, and parallel processing of adjacent frame pairs can be introduced to improve scalability.

**Figure 14:**
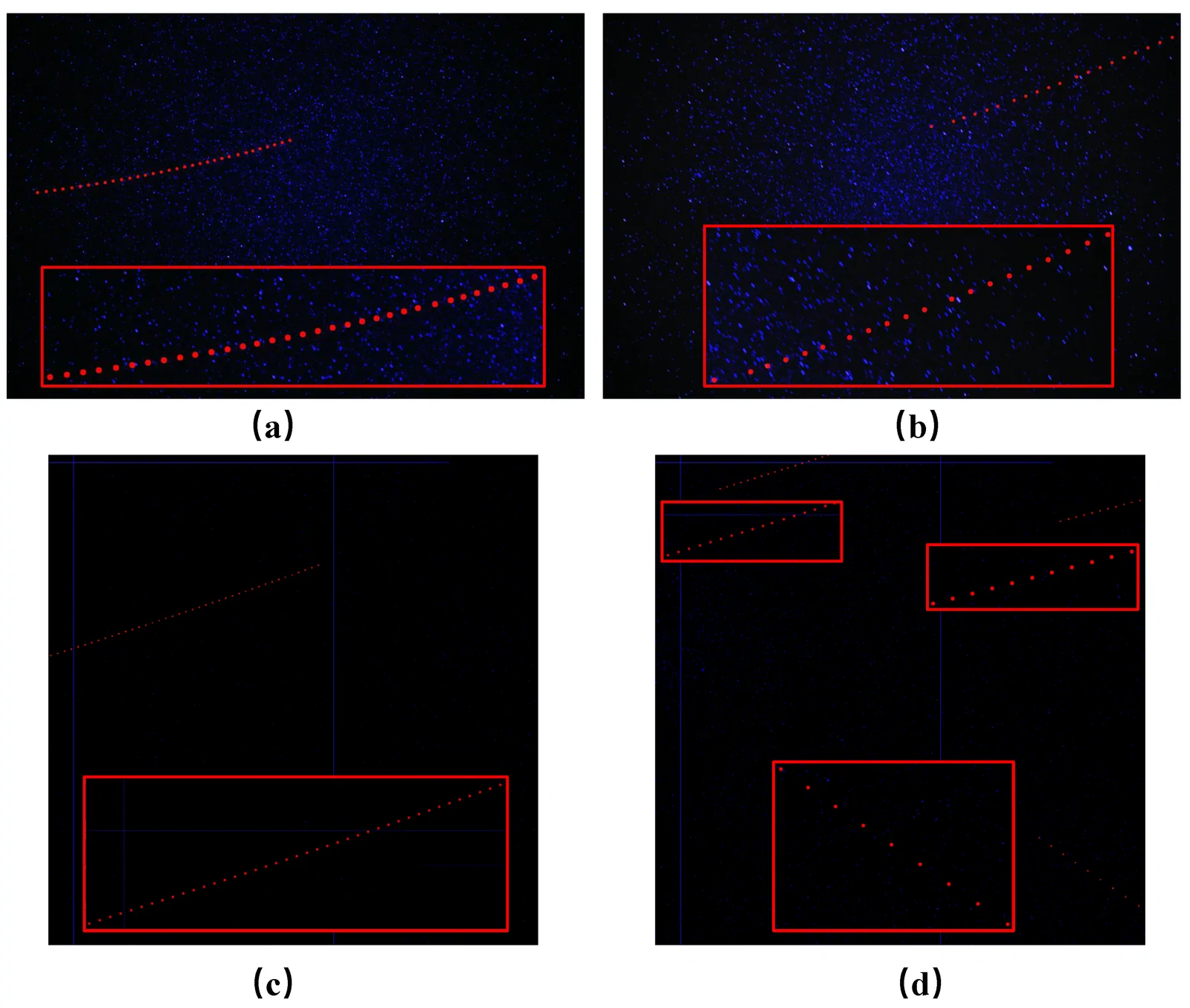
Illustration of spatial target trajectories achieved by our proposed method on the four datasets. (Blue points denote background stars and noise, while red points represent spatial targets. Red dashed lines indicate the estimated target trajectories, and red boxes highlight the corresponding target regions. (**a**) Sequence 1; (**b**) Sequence 2; (**c**) Sequence 3; (**d**) Sequence 4).

To further visualize the motion trajectories of the targets, we plotted the detected targets across all frames in the four datasets, as shown in Figure 14. In the figure, blue denotes stars and background noise, while red indicates spatial targets. This visualization provides a clearer representation of the targets’ motion states and their overall trajectories. The detailed frame-by-frame comparisons between the predicted and true trajectories are provided in Table S1 of the Supplementary Material. The table records the centroid coordinates of the detected targets in pixel units and presents the corresponding frame numbers for the four datasets.

### 4.6. Motion Parameter Uncertainty Analysis

Controlled Monte Carlo simulation. To evaluate the reliability of the proposed spectrum phase shift reconstruction under low-SNR conditions, we conducted a controlled Monte Carlo experiment with known displacement ground truth. For each real dataset, image patches were randomly cropped from the corresponding stellar background images. The annotated target masks were used to avoid target-contaminated regions, ensuring that the generated samples mainly contained real stellar background texture. A known non-integer displacement $\left(d_{x},d_{y}\right)$ was then applied to each patch using bicubic interpolation, and Gaussian noise was added according to the average SNR of the corresponding dataset. For each dataset, 100 Monte Carlo trials were generated.

For the sequence 1 and sequence 2 datasets, pairwise displacement estimation was performed directly. For the two low-SNR sequence 3 and sequence 4 datasets, we further adopted the sequence displacement screening and reconstruction strategy used in the proposed method. Specifically, each Monte Carlo sample was extended to a short image sequence with a constant inter-frame displacement. The displacement candidates estimated from adjacent frame pairs were clustered in the displacement space, and the dominant cluster was selected to reconstruct the final motion parameter. Samples whose reconstructed displacement error exceeded 3 pixels were regarded as unreliable estimates and were counted in the failure rate. The displacement estimation error is defined as

(51) $$e_{i}=\left[\begin{array}{c} {\hat{d}}_{x,i}-d_{x,i}\\ {\hat{d}}_{y,i}-d_{y,i} \end{array}\right]$$

where $\left(d_{x,i},d_{y,i}\right)$ and $\left({\hat{d}}_{x,i},{\hat{d}}_{y,i}\right)$ denote the ground-truth and estimated displacement of the *i*-th Monte Carlo sample, respectively. The Euclidean displacement error is computed as

(52) $$e_{i}^{L2}=\sqrt{{\left({\hat{d}}_{x,i}-d_{x,i}\right)}^{2}+{\left({\hat{d}}_{y,i}-d_{y,i}\right)}^{2}}$$

the motion distance and direction are defined as

(53) $$d_{i}=\sqrt{d_{x,i}^{2}+d_{y,i}^{2}}$$

(54) $$\;\theta_{i}=atan2\left(d_{y,i},d_{x,i}\right)$$

we report the root mean square error (RMSE), the 95th percentile error ($P_{95}$) and the mean absolute angular error(MAE_*θ*_).

As shown in Table 9, the proposed spectrum phase shift reconstruction achieves sub-pixel displacement accuracy on the sequence 1 and sequence 2 datasets. Although the sequence 3 and sequence 4 datasets have lower SNR, the sequence displacement screening and reconstruction strategy effectively suppresses unstable peak responses caused by noise and interpolation errors. For sequence 3, 96 out of 100 trials passed the reliability criterion, with an RMSE of 0.098 pixels and an angular error of 0.105° on valid estimates. For sequence 4, all 100 trials were successfully reconstructed, with an RMSE of 0.110 pixels and an angular error of 0.110°. These results indicate that the proposed method can provide reliable motion parameter estimates under low-SNR stellar backgrounds when combined with sequence-level displacement screening.

It should be noted that the spectrum phase shift reconstruction does not assume that every single-frame phase correlation result is reliable. Instead, unreliable displacement peaks are explicitly rejected through sequence-level screening. Therefore, the reconstructed motion parameters are used as robust constraints for the subsequent directional track association rather than as unfiltered single-frame measurements.

Real-sequence motion error. To further evaluate the reliability of the estimated motion parameters on real image sequences, we compared the inter-frame motion derived from the predicted trajectories with those computed from the annotated target masks. The annotated centroid coordinates in consecutive frames were used as the ground-truth motion reference. The Euclidean displacement error, motion distance, and direction follow the definitions in Equations (46)–(48). We report the mean absolute distance error MAE_*d*_. 

As shown in Table 10, the estimated motion directions are highly consistent with the annotated trajectories on all real sequences. The mean absolute angular errors are below $0.5^{\circ }$ for all datasets, indicating that the proposed motion estimation strategy can provide reliable directional constraints for subsequent trajectory association. In terms of displacement magnitude, the sequence 1 and sequence 3 datasets achieve relatively small errors, with RMSE_*L*2_ values of 1.639 pixels and 0.725 pixels, respectively. The errors on sequence 2 and sequence 4 are larger because these sequences contain faster inter-frame motion and, in the case of sequence 4, multiple target trajectories. Nevertheless, the estimated motion parameters remain within the tolerance range used in the proposed directional track association module. These real-sequence results further confirm that the motion parameters estimated by the spectrum phase shift screening reconstruction are sufficiently stable for guiding target trajectory association.

Ablation study on spectral leakage and sub-pixel peak localization. To analyze the influence of spectral leakage and non-integer displacement interpolation errors, we conducted an ablation study on the phase-correlation-based displacement estimation module. The evaluation was performed on the controlled Monte Carlo samples from the sequence 1 and sequence 2 datasets, where stable single-frame correlation peaks can be obtained. This setting allows the effects of windowing and peak localization to be isolated from the sequence-level screening process. Four variants were compared: integer peak localization, integer peak localization with a Hann window, sub-pixel peak localization, and sub-pixel peak localization with a Hann window. For the integer peak variant, the displacement was directly obtained from the maximum response position in the reconstructed correlation map. For the sub-pixel variant, a local quadratic interpolation was applied around the maximum peak. Given the peak position (x,y) in the correlation response *r*, where *x* and *y* denote the horizontal and vertical coordinates, respectively. The sub-pixel offsets along the x- and y-directions are estimated as

(55) $$\Delta x=\frac{1}{2}\frac{r(x-1,y)-r(x+1,y)}{r(x-1,y)-2r(x,y)+r(x+1,y)},$$

(56) $$\Delta y=\frac{1}{2}\frac{r(x,y-1)-r(x,y+1)}{r(x,y-1)-2r(x,y)+r(x,y+1)}.$$

the refined peak coordinate is then given by

(57) $$\left(x^{sub},y^{sub}\right)=\left(x+\Delta x,y+\Delta y\right).$$

the Hann window was applied before Fourier transformation to reduce boundary discontinuities and suppress spectral leakage.

As shown in Table 11, sub-pixel peak localization substantially improves the accuracy of phase-correlation-based displacement estimation under non-integer shifts. Compared with integer peak localization, sub-pixel refinement reduces RMSE_*L*2_ from 0.430 pixels to 0.164 pixels and decreases the angular error from 0.242° to 0.096°. When the Hann window is further introduced, RMSE_*L*2_ is slightly reduced to 0.160 pixels and the angular error decreases to 0.093°. These results indicate that sub-pixel peak refinement is the dominant factor in reducing non-integer displacement errors, while the Hann window provides additional stabilization by suppressing spectral leakage. Therefore, both operations are retained in the final implementation.

### 4.7. Ablation Study of Key Modules

To further evaluate the contribution of each processing stage, a module-removal ablation study was conducted on the four datasets. In each variant, one key module was removed while the remaining pipeline was kept unchanged. The evaluation metrics include object-level precision (Pre), object-level recall (Re), and pixel-level false alarm rate (Fa). For the variant without image difference fusion, the number of candidate connected components increases dramatically; therefore, the top 300 components in each frame were retained and a 30-min time budget was used for the directional association stage.

As shown in Table 12, each module contributes to the final detection performance. Removing the bright star removal module results in a significant decrease in object-level precision due to the interference caused by abundant residual bright stars. Removing the connected domain processing module has a relatively limited impact on the overall performance, but still leads to a certain degradation in precision and an increase in false alarms, demonstrating its effectiveness in suppressing noise regions with morphological characteristics inconsistent with real targets. When the spectrum phase shift screening and reconstruction module is replaced with a block-matching-based motion estimator, the performance drops significantly on all four datasets. The recall decreases to 17.4%, 28.6%, 35.7%, and 9.8% on sequence 1-sequence 4, respectively, indicating that many dim targets are missed. This is because block matching relies on local spatial similarity and is easily disturbed by dense stellar interference and weak target responses. In contrast, the proposed module reconstructs stable motion parameters through multi-peak screening and sequence-level consistency analysis, providing more reliable constraints for subsequent fusion and association. The image difference fusion coarse processing module plays an important role in suppressing weak stellar interference and preserving target responses. Finally, removing the directional track association module leads to more false trajectories, further demonstrating its effectiveness in improving detection reliability.

### 4.8. Parameter Sensitivity Analysis

The parameters in this paper include the adaptive threshold *T*, area threshold *A*, aspect ratio threshold *R*, compactness threshold *C*, point distance threshold $t_{d}$, direction threshold $T_{a}$ and distance threshold $T_{d}$. The settings of these parameters have a certain impact on the detection performance of the model. The adaptive threshold *T* determines the enhancement level of faint stars and space objects. A smaller threshold *T* retains more pixels in the image, while a larger *T* removes more pixels. The parameter *k* decides the size of the adaptive threshold *T*, and based on experience, it is set to k=2.5. The area threshold *A*, aspect ratio threshold *R* and compactness threshold *C* can eliminate stars and noise that do not meet the criteria in the post-processing of images. They are empirically set to 5<A<1000, 0.3<R<3.5, and C<0.3. The neighbourhood radius ρ is used only for local non-maximum suppression in the correlation map to avoid repeatedly selecting neighbouring responses around the same peak, rather than as a physical target-association threshold. In this study, ρ=25 pixels is selected according to the observed correlation-peak width and the separation between background and target-related peaks in the displacement space. The point distance threshold $t_{d}$ controls the similarity grouping of displacement candidates. Based on the real-sequence motion error analysis in Table 10, the largest 95th percentile displacement error is 10.536 pixels; therefore, $t_{d}=15$ pixels provides sufficient tolerance for motion-estimation errors while reducing the risk of merging different motion clusters. The direction threshold $T_{a}$ and distance threshold $T_{d}$ determine the size of the range for directional track association. When $T_{a}$ and $T_{d}$ are set to larger values, the search range for tracks is broader, increasing the likelihood of detecting targets but also resulting in more false alarms. Conversely, when $T_{a}$ and $T_{d}$ are set to smaller values, the search range is narrower, leading to more precise target detection but potentially causing missed detections. Therefore, $T_{a}=20^{{}^{\circ}}$ and $T_{d}=30$ are set based on experience to better balance false alarms and missed detections.

**Figure 15:**
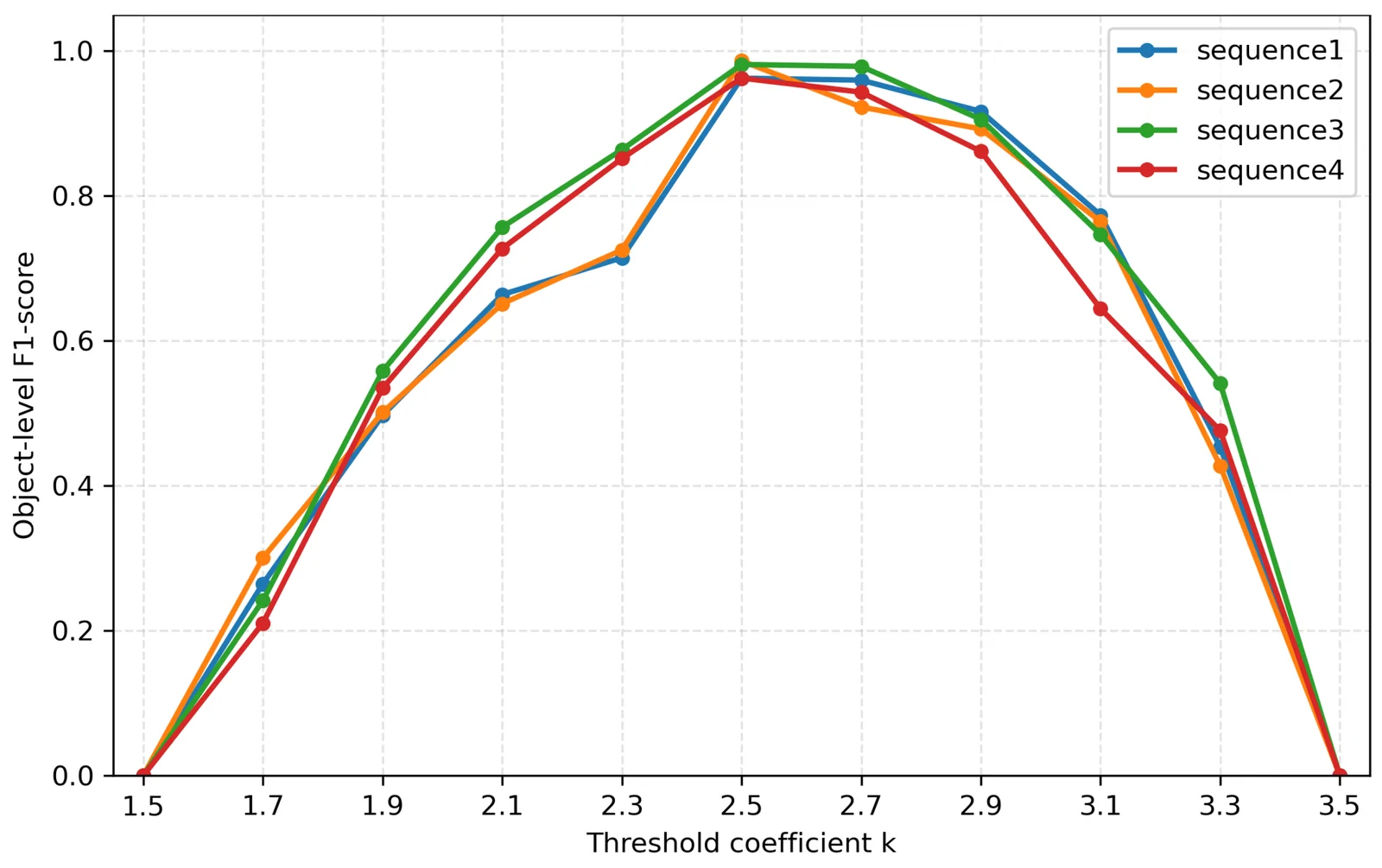
Sensitivity analysis of the adaptive threshold coefficient *k* on four image datasets.

To evaluate the influence of the adaptive threshold coefficient *k* on the proposed method, a parameter sensitivity analysis was conducted on the four datasets. The coefficient *k* was varied from 1.5 to 3.5 with an interval of 0.2. For each value of *k*, the complete target detection pipeline was executed. Figure 15 plots the F1-score against the adaptive threshold coefficient *k*, illustrating the variation in detection performance under different parameter settings and thereby demonstrating the influence of *k* on the overall performance of the proposed method.

The sensitivity curves are presented in Figure 15. When *k* is too small, an excessive number of stellar background components survive the thresholding operation. Although this may help retain more candidate targets, the increased false alarms interfere with subsequent spectral phase-shift estimation and directional trajectory association, ultimately raising the false-positive rate and compromising overall detection performance. Conversely, when *k* is too large, the threshold becomes overly stringent. Weak target responses and useful stellar components may be suppressed, leading to an insufficient number of reliable references for motion-parameter estimation. This can result in inaccurate motion estimates and fragmented target trajectories. The results indicate that the proposed method maintains stable performance for *k* in the range of 2.5 to 2.9. In particular, k=2.5 provides a favorable trade-off between false-alarm suppression and target retention while maintaining a competitive F1-score. Accordingly, k=2.5 is adopted as the default parameter setting in this study.

## 5. Conclusions

This paper proposes a method for detecting dim space targets and predicting trajectories in dense star backgrounds. First, bright stars are filtered out through inter-frame image registration and connected domain post-processing to simplify the background. Next, it designs a spectrum phase shift screening reconstruction method, which estimates the faint star and target motion parameters based on the inter-frame phase differences to obtain a reconstructed multi-impulse function sequence. Following this, an image differential fusion coarse processing module is designed to generate candidate targets. Finally, a directional track association is devised based on the centers and motion parameters of the candidate targets, narrowing the association range to a sector area, which eliminates unnecessary false alarms while detecting the actual target trajectories. Experiments show that this method exhibits good robustness in detecting targets of varying brightness levels, improving the accuracy of faint target detection and effectively reducing false alarms.

Nevertheless, it should be noted that when the image sequence contains large rotation, significant viewpoint change, or abrupt target motion, the registration method used in the bright-star removal module may become insufficient, leading to residual stellar structures and degraded candidate extraction performance. In such cases, more robust geometric alignment methods should be introduced. Our method has significant room for improvement in detection performance. Future research directions include enhancing the signal-to-noise ratio of faint targets and overall detection efficiency, as well as conducting more comprehensive scalability evaluations under larger image sizes and higher frame rates.

## Figures and Tables

**Table 1:** Details of four space target datasets.

Datasets	Frame	Image Resolution	Average *SNR*	Approx. Star	Scene Description
				**Density (Stars/MP)**	
sequence 1	51	3680×2456	2.662	198.1 stars/MP	Dense stars The target speed is 52 pixels/frame
sequence 2	26	3680×2456	1.389	125.8 stars/MP	Dense stars The target speed is 78 pixels/frame
sequence 3	50	6144×6144	0.715	40.5 stars/MP	Dense stars The target speed is 89 pixels/frame
sequence 4	50	6144×6144	0.646	85.0 stars/MP	Dense stars The target speed is 189/101/89 pixels/frame

**Table 2:** Parameter settings of different comparison methods.

Method	Parameter Settings
STLDM	Threshold parameter k=5∼15
CTSTC	f=3, $\lambda_{L}=30$, μ=0.01, η=1.0
MFSTPT	Patch size: 60×60, step: 60, $\lambda =1/\sqrt{\max\left(n_{1},n_{2}\right)n_{3}}$
MSAMNet	Adagrad optimizer, initial learning rate: 0.01, StepLR scheduler with decay interval of 50 epochs and decay factor of 0.1, α=0.75, γ=2.
TESNet	Temporal window: 5 frames, training epochs: 100, batch size: 4, Adam optimizer with momentum of 0.8, initial learning rate: $1\times 10^{-4}$, cosine annealing scheduler.
Proposed	k=2.5, $T_{a}=20^{{}^{\circ}}$, $T_{d}=30$ pixels.

**Table 3:** Comparison of different methods evaluated by Pre, Re and $F_{a}$.

Method	Sequence 1			Sequence 2			Sequence 3			Sequence 4		
	*Pre*	*Re*	*F_a_*	*Pre*	*Re*	*F_a_*	*Pre*	*Re*	*F_a_*	*Pre*	*Re*	*F_a_*
STLDM	24.2%	48.6%	$6.23\times 10^{-4}$	26.6%	55.7%	$5.91\times 10^{-4}$	31.5%	82.6%	$9.24\times 10^{-6}$	35.6 %	85.0%	** $2.46\times 10^{-6}$ **
CTSTC	Failed	Failed	Failed	Failed	Failed	Failed	53.1%	54.6%	$3.18\times 10^{-6}$	58.8%	60.1%	$1.09\times 10^{-6}$
MFSTPT	43.7%	41.5%	$3.16\times 10^{-5}$	39.6%	42.8%	$9.22\times 10^{-5}$	Failed	Failed	Failed	Failed	Failed	Failed
MSAMNet	Failed	Failed	Failed	Failed	Failed	Failed	73.6%	86.5%	$3.36\times 10^{-6}$	78.2%	67.7%	$3.54\times 10^{-6}$
TESNet	82.3%	78.6%	$5.75\times 10^{-6}$	84.6%	77.2%	$4.68\times 10^{-6}$	75.3%	76.3%	$3.14\times 10^{-6}$	73.9%	71.4%	$3.87\times 10^{-6}$
**Proposed**	**98.7%**	**93.9%**	** $4.48\times 10^{-6}$ **	**97.3%**	**100%**	** $3.91\times 10^{-6}$ **	**96.7%**	**99.6%**	** $2.44\times 10^{-6}$ **	**95.8%**	**96.7%**	** $2.37\times 10^{-6}$ **

*Note:* Failed indicates that the corresponding method was executed but failed to obtain effective target detection results because excessive false alarms overwhelmed the real target responses.

**Table 4:** Statistical results of different methods on $pAUC_{ROC}\;\left(\times 10^{-6}\right)$ and PR-$AUC\;\left(\times 10^{-1}\right)$.

Method	Sequence 1		Sequence 2		Sequence 3		Sequence 4	
	*pAUC_ROC_*	*PR-AUC*	*pAUC_ROC_*	***PR-AUC***	*pAUC_ROC_*	*PR-AUC*	*pAUC_ROC_*	*PR-AUC*
STLDM	N/A	1.979	N/A	2.118	N/A	3.440	N/A	4.335
CTSTC	N/A	Failed	N/A	Failed	N/A	4.730	N/A	5.672
MFSTPT	N/A	2.821	N/A	2.236	N/A	Failed	N/A	Failed
MSAMNet	N/A	Failed	N/A	Failed	N/A	8.251	N/A	7.720
TESNet	N/A	8.678	N/A	8.443	N/A	7.967	N/A	7.849
**Proposed**	**9.441**	**9.891**	**9.529**	**9.614**	**9.666**	**9.803**	**9.457**	**9.908**

*Note:* N/A indicates that the corresponding $pAUC_{ROC}$ value is not reported, since only the proposed method is evaluated by ROC curves under a unified false alarm interval due to the significant difference in false alarm levels among different methods.

**Table 5:** Comparison with other state-of-the-art methods on IoU.

Method	*IoU*			
	Sequence 1	Sequence 2	Sequence 3	Sequence 4
STLDM	0.115	0.143	0.398	0.355
CTSTC	Failed	Failed	0.101	0.090
MFSTPT	0.036	0.032	Failed	Failed
MSAMNet	Failed	Failed	0.547	0.480
TESNet	0.443	0.451	0.717	0.672
**Proposed**	**0.672**	**0.718**	**0.885**	**0.748**

*Note:* Failed indicates that the corresponding method was executed but failed to obtain valid target detection results; therefore, the IoU metric could not be calculated.

**Table 6:** The average deviation error of associated tracks for different methods (in pixels).

Method	Sequence 1	Sequence 2	Sequence 3	Sequence 4		
				Target1	Target2	Target3
STLDM	3.06	4.50	2.47	3.13	3.19	1.12
CTSTC	Failed	Failed	2.30	2.27	2.28	2.31
MFSTPT	3.25	2.86	Failed	Failed	Failed	Failed
MSAMNet	Failed	Failed	**0.18**	1.34	1.56	3.14
TESNet	**0.76**	2.71	0.33	1.25	1.13	2.85
**Proposed**	0.81	**2.57**	0.26	**1.22**	**1.06**	**2.05**

*Note:* Failed indicates that the corresponding method was executed but failed to obtain valid target detection results; therefore, the average deviation error could not be calculated.

**Table 7:** The average computational efficiency (in seconds) of different methods.

Time(s)	STLDM	CTSTC	MFSTPT	MSAMNet	TESNet	Proposed
sequence 1	5.24	Failed	6.43	Failed	**1.04**	2.32
sequence 2	6.02	Failed	6.69	Failed	**0.95**	2.24
sequence 3	14.10	18.34	Failed	**2.84**	3.07	7.51
sequence 4	14.42	18.02	Failed	**2.91**	2.97	7.38

*Note:* Failed indicates that the corresponding method was executed but failed to obtain valid target detection results; therefore, the average computational efficiency could not be calculated.

**Table 8:** Average running time of each module in the proposed method (in seconds).

Dataset	BSR-CDP	SPSR	IDFCP	DTA	Total
sequence 1	0.4614	1.8274	0.0665	0.0684	2.4237
sequence 2	0.4937	2.1318	0.0581	0.0828	2.7663
sequence 3	1.0071	7.7168	0.4722	1.0900	10.2861
sequence 4	1.7239	7.4546	0.4100	0.4882	10.0766

*Note:* The stage-wise runtime was measured using ten consecutive frames containing valid target trajectories from each dataset. Therefore, these results are intended to analyze the computational bottlenecks of the proposed method and are not directly comparable to the full-sequence average runtime reported in Table 7.

**Table 9:** Uncertainty analysis of motion parameter estimation under controlled Monte Carlo simulations.

Dataset	SNR	Trials	Valid Trials	RMSE_*L*2_ (Pixel)	*P*_95_ (Pixel)	MAE_*θ*_ (°)
sequence 1	2.66	100	100	0.176	0.255	0.088
sequence 2	1.39	100	100	0.143	0.222	0.098
sequence 3	0.72	100	96	0.098	0.163	0.105
sequence 4	0.65	100	100	0.110	0.197	0.110

**Table 10:** Motion parameter errors on real image sequences.

Dataset	SNR	Pairs	RMSE_*L*2_	MAE_*d*_ (Pixel)	MAE_*θ*_ (°)	*P*_95_ (Pixel)
sequence 1	2.66	32	1.639	0.998	0.500	3.606
sequence 2	1.39	19	4.110	2.542	0.498	10.536
sequence 3	0.72	40	0.725	0.404	0.139	1.414
sequence 4	0.65	34	3.670	2.597	0.284	6.997

**Table 11:** Ablation study on spectral leakage suppression and sub-pixel peak localization.

Variant	RMSE_*L*2_	*P*_95_	MAE_*θ*_	Failure Rate
	**(Pixel)**	**(Pixel)**	**(°)**	
Integer peak	0.430	0.614	0.242	0%
Integer peak + Hann window	0.429	0.614	0.241	0%
Sub-pixel peak	0.164	0.253	0.096	0%
Sub-pixel peak + Hann window	0.160	0.253	0.093	0%

**Table 12:** Ablation study of different modules evaluated by object-level Pre, object-level Re, and pixel-level $F_{a}$.

Method	Sequence 1			Sequence 2			Sequence 3			Sequence 4		
	*Pre*	*Re*	*Fa*	*Pre*	*Re*	*Fa*	*Pre*	*Re*	*Fa*	*Pre*	*Re*	*Fa*
Full method	98.7%	93.9%	$4.48\;\times \;10^{-6}$	97.3%	100.0%	$3.91\;\times \;10^{-6}$	95.8%	96.7%	$2.37\;\times \;10^{-6}$	96.7%	99.6%	$2.44\;\times \;10^{-6}$
w/o Bright star removal	56.5%	79.9%	$9.71\;\times \;10^{-6}$	47.6%	86.3%	$9.40\;\times \;10^{-6}$	66.1%	84.0%	$8.84\;\times \;10^{-6}$	30.3%	87.3%	$8.09\;\times \;10^{-6}$
w/o Connected domain processing	88.8%	90.1%	$5.62\times 10^{-6}$	87.6%	97.2%	$4.98\times 10^{-6}$	85.3%	96.2%	$3.53\times 10^{-6}$	86.5%	98.7%	$3.69\times 10^{-6}$
w/o Spectrum phase shift reconstruction ^†^	38.5%	17.4%	$1.49\;\times \;10^{-6}$	41.4%	28.6%	$1.94\;\times \;10^{-6}$	57.3%	35.7%	$1.65\;\times \;10^{-6}$	64.7%	9.8%	$7.82\;\times \;10^{-7}$
w/o Image difference fusion	20.4%	21.7%	$6.8\;\times \;10^{-5}$	18.7%	15.3%	$6.4\;\times \;10^{-5}$	14.3%	18.5%	$5.3\;\times \;10^{-5}$	15.7%	22.2%	$4.7\;\times \;10^{-5}$
w/o Directional track association	80.4%	89.1%	$5.02\;\times \;10^{-6}$	81.7%	82.4%	$4.10\;\times \;10^{-6}$	86.8%	88.9%	$3.47\;\times \;10^{-6}$	83.9%	80.7%	$3.56\;\times \;10^{-6}$

*Note:* For the variant without image difference fusion, the top 300 connected components were retained in each frame and a 30-min time budget was used for directional track association. ^†^ indicates that the spectrum phase shift reconstruction module is replaced with a block-matching-based motion estimator.

## Data Availability

The data presented in this study are available on request from the corresponding author.

## Supplementary Materials

The following supporting information can be downloaded at: https://www.mdpi.com/article/10.3390/s26165294/s1, Table S1: Comparative plots of predicted versus true trajectory coordinates by our method across the four datasets.
