# Supplementary material for: A Dim Space Target Detection and Track Association Method for Dense Stellar Backgrounds
Source: Sensors (Basel). 2026 Aug 21;26(16):5294. doi: 10.3390/s26165294 (PMC13517305; doi:10.3390/s26165294)
Supplement: Supplementary file 1 [file sensors-26-05294-s001.zip › sensors-4404331-supplementary.pdf]

Table S1 presents the frame-by-frame comparison between the predicted trajectories and the corresponding ground-truth trajectories obtained by our method across the four datasets. The centroid positions of spatial targets are recorded in pixel coordinates, where x_coord and y_coord denote the horizontal and vertical coordinates, respectively, and $frame\_number$ indicates the corresponding image frame index. The comparison provides detailed trajectory localization results and demonstrates the consistency between the estimated and true target motion paths.

Table S1: Comparative plots of predicted versus true trajectory coordinates by our method across the four datasets.

sequence1						sequence2						sequence3						sequence4					
True Trajectory			Predicted Trajectory			True Trajectory			Predicted Trajectory			True Trajectory			Predicted Trajectory			True Trajectory			Predicted Trajectory		
x_coord	y_coord	frame_number	x_coord	y_coord	frame_number	x_coord	y_coord	frame_number	x_coord	y_coord	frame_number	x_coord	y_coord	frame_number	x_coord	y_coord	frame_number	x_coord	y_coord	frame_number	x_coord	y_coord	frame_number
11	1165	17				2091	721	1	2091	721	1	29	2516	10	29	2516	10	4818	4804	1	4816	4802	1
71	1157	18				2232	690	2	2233	689	2	115	2489	11	115	2489	11	4966	4905	2	4966	4905	2
194	1143	19	194	1143	19	2300	666	3	2306	664	3	201	2462	12	201	2462	12	5118	5009	3	5118	5009	3
250	1135	20	250	1135	20	2370	642	4	2370	642	4	286	2435	13	286	2435	13	5270	5113	4	5272	5114	4
305	1127	21	304	1127	21	2445	617	5	2445	617	5	372	2408	14	372	2408	14	5426	5220	5	5426	5220	5
356	1120	22	356	1120	22	2515	592	6	2514	593	6	458	2380	15	458	2380	15	5585	5328	6	5583	5327	6
412	1112	23	413	1112	23	2619	555	7	2611	558	7	543	2353	16	543	2353	16	5742	5436	7	5742	5436	7
467	1103	24	467	1103	24	2695	527	8	2700	526	8	629	2326	17	628	2326	17	5903	5546	8	5902	5545	8
520	1095	25	519	1095	25	2766	502	9	2765	501	9	714	2298	18	713	2299	18	6065	5656	9	6063	5655	9
573	1087	26	572	1087	26	2840	474	10	2839	474	10	799	2271	19	799	2271	19						
626	1078	27	626	1078	27	2913	447	11	2911	447	11	884	2244	20	883	2244	20						
679	1069	28	676	1069	28	3017	405	12	3011	407	12	968	2216	21	968	2216	21	5084	830	1	5084	830	1
731	1060	29	730	1060	29	3092	376	13	3091	376	13	1053	2189	22	1053	2189	22	5184	804	2	5182	805	2
783	1051	30	783	1051	30	3167	345	14	3167	346	14	1137	2161	23	1137	2161	23	5281	779	3	5281	779	3
834	1041	31	834	1041	31	3242	314	15	3242	315	15	1221	2134	24	1221	2134	24	5380	753	4	5382	752	4
882	1031	32	881	1032	32	3320	283	16	3319	283	16	1306	2106	25	1305	2106	25	5481	727	5	5481	727	5
930	1022	33	932	1021	33	3395	252	17	3393	252	17	1390	2078	26	1390	2078	26	5579	701	6	5580	701	6
983	1011	34	983	1012	34	3474	218	18	3471	220	18	1474	2050	27	1474	2050	27	5681	674	7	5680	674	7
1033	1001	35	1033	1001	35	3552	185	19	3547	187	19	1558	2022	28	1557	2022	28	5780	648	8	5779	648	8
1083	990	36	1083	990	36	3628	152	20	3626	154	20	1642	1994	29	1642	1994	29	5882	621	9	5881	621	9
1131	979	37	1132	979	37							1725	1966	30	1725	1966	30	5981	595	10	5978	596	10
1183	969	38	1182	969	38							1809	1938	31	1809	1938	31	6079	569	11	6079	569	11
1230	958	39	1230	958	39							1892	1910	32	1892	1910	32						
1278	947	40	1278	949	40							1975	1882	33	1975	1882	33						
1323	937	41	1322	937	41							2059	1853	34	2059	1853	34	815	423	1	813	424	1
1370	925	42	1369	925	42							2142	1825	35	2142	1825	35	898	398	2	902	397	2
1415	914	43	1415	914	43							2226	1796	36	2225	1796	36	987	371	3	985	371	3
1473	901	44	1472	902	44							2308	1767	37	2308	1767	37	1070	346	4	1073	345	4
1520	889	45	1524	888	45							2391	1739	38	2392	1738	38	1161	318	5	-	-	5
1566	876	46	1566	877	46							2475	1709	39	2475	1709	39	1245	292	6	1244	392	6
1612	864	47	1611	864	47							2558	1680	40	2558	1680	40	1331	266	7	1331	265	7
1658	851	48	1659	851	48							2641	1651	41	2641	1651	41	1415	239	8	1416	239	8
1705	838	49	1705	838	49							2724	1622	42	2724	1622	42	1502	212	9	1501	212	9
1754	825	50	1754	824	50							2807	1593	43	2807	1592	43	1586	186	10	1589	185	10
1802	810	51	1802	810	51							2890	1563	44	2890	1563	44	1670	160	11	1661	162	11
												2973	1533	45	2973	1533	45	1757	132	12	1756	132	12
												3057	1503	46	3057	1503	46	1841	105	13	1841	105	13
												3140	1474	47	3139	1474	47	1927	78	14	1927	78	14
												3223	1443	48	3223	1443	48	2006	52	15	2009	52	15
												3306	1413	49	3306	1413	49	2095	24	16	2094	24	16
												3390	1383	50	3389	1383	50	2168	0	17	2168	1	17
